# K^+^ channel blockade limits AF and suppresses phase 3 EADs by slowing repolarization in an electromechanical cell computational model

**DOI:** 10.3389/fphys.2025.1704051

**Published:** 2026-01-22

**Authors:** Fazeelat Mazhar, Stefano Severi, Chiara Bartolucci

**Affiliations:** 1 Department of Pharmacology, University of California, Davis, CA, United States; 2 Department of Electrical, Electronic and Information Engineering “Guglielmo Marconi”, University of Bologna, Cesena, Italy

**Keywords:** anti-arrhythmic, atrial fibrillation, atrial-specific blockers, contraction, electromechanical coupling, human atrial cells, *in silico* modeling

## Abstract

**Purpose:**

Selective inhibition of atrial proarrhythmicity can be therapeutic for reducing the atrial fibrillation (AF) burden. Atrial-selective K^+^-channel blockade (mainly Kv1.5 and Kv4.3 channels conducting the sustained I_Kur_ and transient I_to_ outward currents) promises to suppress AF with a favorable benefit-to-harm ratio. The mechanisms underlying the efficacy of K^+^ channel blockade under arrhythmic conditions and its association with electrophysiological and contractile remodeling in AF remain to be investigated.

**Methods:**

Using our electromechanically coupled model MBS2023, we have simulated the effects of 4-aminopyridine (4-AP) and AVE0118 at different basic cycle lengths (2–0.25s). We have dissociated the primary and secondary responses to determine the drug’s underlying mechanisms of action. We have analyzed the effects of K^+^-channel blockers under arrhythmogenic conditions induced by either forward excitation-contraction coupling (ECC) or mechano-calcium feedback.

**Results:**

At the basal rate, the voltage-mediated increase in I_Kr_ induced by 4-AP shortens the action potential duration (APD) under sinus rhythm (SR), whereas a surge in I_CaL_ prolongs APD under AF. 4-AP can exacerbate the vulnerability to phase 2 early afterdepolarizations (EADs) by slowing repolarization and prolonging myofilament activation. K^+^-channel blockade can decimate the susceptibility of delayed afterdepolarizations (DADs) by eliminating the cytosolic Ca^2+^ overload. The slowing of repolarization induced by 4-AP can suppress the reopening of Na^+^ channels during phase 3 EADs.

**Conclusion:**

In both types of EAD, a shorter, Ca^2+^-desensitized sarcomere can reduce the propensity for AF in the model. In general, K^+^ channel blockade has anti-arrhythmic potential to suppress phase 3 EADs by slowing repolarization.

## 1 Introduction

Atrial fibrillation (AF) represents the most prevalent sustained cardiac arrhythmia. Its prevalence is projected to at least double in the coming decades, primarily due to population aging, the growing burden of comorbidities, increased clinical awareness, and advances in diagnostic technologies (Du et al., 2017; Van Gelder et al., 2024). Long-term rhythm control in AF can be achieved through electrical cardioversion, catheter ablation, or pharmacological therapy with anti-arrhythmic drugs. However, current therapeutic strategies remain limited by suboptimal efficacy and the risk of adverse effects, including proarrhythmia with the potential to trigger life-threatening ventricular arrhythmias (Ang et al., 2020).

**FIGURE 1:**
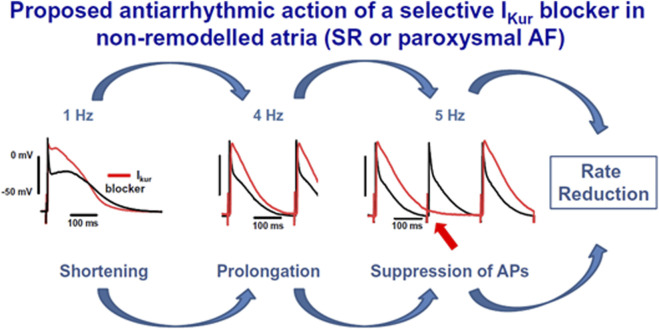
Proposed antiarrhythmic action of a selective $I_{\mathrm{Kur}}$ block in non-remodeled atria. The action potential duration (APD) gets abbreviated at a normal frequency of 1 Hz, is prolonged at 4 Hz, and onwards in the presence of the $I_{\mathrm{Kur}}$ -specific block (in red). The excitability of the AP gets suppressed because of drug-induced APD prolongation. The figure is obtained from Ravens and Odening (2017), with permission.

Atrial-selective block targets $K^{+}$ channels with predominant atrial expression, thereby preventing ventricular repolarization prolongation. Kv1.5 channels, which conduct the sustained outward current $\left(I_{\mathrm{Kur}}\right)$ in human atrial myocytes, represent a key determinant of this atrial selectivity (Ravens and Wettwer, 2011). The anticipated effect of blocking an outward repolarizing current is to lengthen the action potential duration (APD) and, hence, to limit reentry by prolonging the effective refractory period (ERP). However, in human atrial tissue under sinus rhythm (SR), a low concentration of 4-aminopyridine (4-AP), an $I_{\mathrm{Kur}}$ -selective blocker, elevates the plateau potential and shortens the APD instead of lengthening it (Wettwer et al., 2004). The modulation of APD and ERP by selective $I_{\mathrm{Kur}}$ blockade strongly depends on the pacing frequency. MK-0448, another $I_{\mathrm{Kur}}$ -selective blocker, when applied on healthy human subjects, shortens the ERP at 1 Hz (Loose et al., 2014), but it does not produce any electrophysiological effect on atrial refractoriness at 2.5 Hz (Pavri et al., 2012). XEN-D0103, an alternative and highly $I_{\mathrm{Kur}}$ -specific blocker, abbreviated the APD at 1 Hz but prolonged it and suppressed excitability at higher pacing frequencies ( > 3 Hz) in human atrial tissue from patients in SR (Ford et al., 2016), also shown in Figure 1. Instead, in tissue from AF patients, $I_{\mathrm{Kur}}$ blocking effect was the prolongation of APD and ERP for all frequencies (Ford et al., 2016).

Based on these results, the $I_{\mathrm{Kur}}$ -selective drug block was proposed to reverse the AF-induced burden at AF-relevant rates. However, the results of phase 2 clinical trials for XEN-D0103 suggest no significant reduction in AF burden, with no serious adverse effects (Shunmugam et al., 2018). Nevertheless, the complex primary and secondary effects of $I_{\mathrm{Kur}}$ channel blockade have not been fully elucidated and understood, thereby limiting the potential for these agents to be used in combination with other atrial-specific drugs or as genotype-specific AF treatments. Of note, computational analysis has not helped much so far, given that the available computational models were not able to accurately reproduce the counterintuitive rate-dependent effects of $I_{\mathrm{Kur}}$ block on APD (as shown in the discussion). These observations provided the rationale for conducting this study. In native human atrial cardiomyocytes, drug effects on $K^{+}$ currents, $I_{\mathrm{Kur}}$ and $I_{to}$ , are difficult to distinguish due to the presence of substantial overlap (Ravens and Wettwer, 2011). Accordingly, in this study, we analyzed and compared the effects of low-dose concentrations of the $I_{\mathrm{Kur}}$ -specific blocker 4-AP and the nonspecific agent AVE0118 on atrial electrophysiology and contractility. To this end, we used computational modeling, which offers a powerful and efficient approach for gaining mechanistic insights under both physiological and pathological conditions.

We aim to dissociate the primary and secondary responses by which $K^{+}$ channel blockade affects the electrophysiology and contractility under i) SR and AF conditions and ii) at basal and AF-relevant rates. We have utilized our recently developed electro-mechanical model for human atrial cardiomyocytes, Mazhar–Bartolucci–Severi 2023 (MBS2023) (Mazhar et al., 2024), with some modification. Using the updated model, we incorporated AF-associated alterations through an incremental approach that accounted for electrical remodeling and contractile dysfunction of atrial cardiomyocytes. In particular, intercellular $Ca^{2+}$ handling and sarcomeric protein function were modified in accordance with experimental findings (Belus et al., 2010; Fakuade et al., 2024). The unique aspect of this study is the use of well-calibrated SR and AF electro-mechanical models to investigate the effects of $K^{+}$ -channel blockade on both electrophysiology and inotropic response. We demonstrate how drug-induced plateau enhancement translates into a positive inotropic effect under SR and AF conditions. This integrative approach enables the identification of potential therapeutic targets from both electrical activity and contractile function perspectives.

Using the model, we investigated the role of $K^{+}$ -channel blockade in the initiation and maintenance of AF. To this end, we simulated the protocols to induce afterdepolarization abnormalities, including early afterdepolarizations (EADs) during phases 2 and 3 of the action potential and delayed afterdepolarizations (DADs) during diastole, under both SR and AF conditions. Our analysis indicates that $K^{+}$ -channel block–induced slowing of repolarization may be beneficial in preventing $Na^{+}$ -channel reactivation in the presence of phase 3 EADs, yet may exacerbate vulnerability to phase 2 EADs. In both types of EADs, myofilament desensitization and reduced sarcomere length attenuated the proarrhythmic substrate in the model. Furthermore, $K^{+}$ -channel block diminished susceptibility to $Ca^{2+}$ -induced DADs by alleviating cytosolic $Ca^{2+}$ overload. Collectively, these findings suggest that although $K^{+}$ -channel blockade has anti-arrhythmic potential in suppressing phase 3 EAD–mediated triggered activity, it does not protect against AP– $Ca^{2+}$ –driven phase 2 EADs, which may facilitate tachyarrhythmogenesis.

## 2 Methods

### 2.1 Updates in the electro-mechanical model, MBS2023

MBS2023 is an electromechanically coupled human atrial cardiomyocyte model with a detailed multi-compartmental structure. The model captures the characteristics of the human atrial AP, $Ca^{2+}$ transient (CaT), and active tension (Ta) under physiological conditions, as discussed by Mazhar et al. (2024). In this work, we propose a few updates in the ionic current formulation and in the contractile machinery of the integrated model.

Among the ionic currents, $I_{to}$ formulation in MBS2023 was previously adopted by Nygren et al. (1998), which was based on rabbit experimental data. The rabbit $I_{to}$ underlies an α -subunit encoded by the Kv1.4 gene that differs from the human atrial $I_{to}$ isoform, Kv4.3, in many ways. For instance, Kv1.4 recovers slowly and exhibits frequency dependency; however, these properties are in contrast to those of Kv4.3 (Wang et al., 1999). Accordingly, we updated the formulation of the $I_{to}$ current in the MBS2023 model to reflect the experimental data (Wang et al., 1999; Gao et al., 2005) adapted from the study by Ni et al. (2019). For the $I_{Kr}$ current, the slope for inactivation was reduced to 13 to ensure complete inactivation at positive membrane potentials. In the parent model, the inactivation time constant $\left(I_{\mathrm{Krpatau}}\right)$ of $I_{Kr}$ was derived from rabbit experimental data (Nygren et al., 1998); therefore, we adopted the formulation from the model proposed by Courtemanche et al. (1998), which is based on human atrial experimental data (Wang et al., 1993). The updated $I_{Kr}$ inactivation curve and time constant are shown in Supplementary Figure S1. As a consequence of these changes, the APD rate dependence was disrupted at slow pacing rates. To restore this, the $RyR_{ss}$ inactivation recovery time $\left(RyRtauinact_{ss}\right)$ was made faster from 450 to 12 msec. The maximum and minimum values of $RyR_{ss}$ adaptation ( $RyRa1_{ss}$ and $RyRa2_{ss}$ ) were restored to their original KM2011 values (0.505 and 0.427, respectively). As the model AP has a more positive plateau resulting in more $Ca^{2+}$ in the cytosol, the EC50 value of the calcium-dependent inactivation (CDI) fCa gate in the $I_{\mathrm{CaL}}$ current, KCa , was shifted to the right from 0.65 to 0.68 μ M. Finally, the conductance of $K^{+}$ currents was increased, for instance, $g_{to}$ by 1.5 times and $g_{Kr}$ by 4 times, whereas for $Na^{+}$ currents, it was reduced to 250 m. Overall, all the changes in the updated model are listed in Supplementary Table S1.

In MBS2023, the contraction model cross-bridge (XB) transition rate parameters were kept the same as in the default RDQ model (Regazzoni et al., 2020). Compared to human atrial myofibrils data (Piroddi et al., 2007), the RDQ default parameter setting generates a force that has faster XB cycling (r0) rates and slower kinetics of the thin filament regulatory unit (RU) ( $k_{\mathrm{off}}$ and $k_{\mathrm{basic}}$ ). Therefore, the XB and RU rates were retuned by simulating the experimental protocol, the fast solution-switching protocol (Piroddi et al., 2007), as shown in Supplementary Figure S2. In brief, the protocol proceeds with a $Ca^{2+}$ step from 0.1 to 316 μ M that develops a force with the activation rate, $K_{\mathrm{act}}$ . With high $Ca^{2+}$ , the slack test, when applied, redevelops another force with a transition rate of $K_{tr}$ . By estimating optimal fits for $K_{\mathrm{act}}$ and $K_{tr}$ with the experimental data extrapolated at $37^{o}C$ (Piroddi et al., 2007; Ferrantini et al., 2022), we could recalibrate the XB kinetics, as shown in Supplementary Figures S2, 3. The model parameters re-tuned for this calibration are listed in Table 1. Meanwhile, the Ta–pCa curve was also revisited and was shifted toward the left compared with that in MBS2023 (Supplementary Figure S3 bottom left panel), and this was achieved by re-tuning the RU steady-state parameters μ , kd0 , and γ that decide the asymptotic behavior, EC50, and slope of the curve, respectively. All recalibrated contraction-related parameters are listed in Table 1.

**TABLE 1:** Parameters for the RDQ contraction model calibrated using the fast solution switching protocol under control conditions for human atrial myosin isoform expressing α myofibrils (Piroddi et al., 2007).

Parameter	RDQ **(Default)**	Calibrated	Parameter	RDQ **(Default)**	Calibrated
RU steady state XB kinetics					
γ (−)	12	20	$\mu_{fp}^{0}$ $\left(s^{-1}\right)$	32.653	16.65
$k_{d0}$ (pCa)	6.41	6.29	$\mu_{fp}^{1}$	0.773	0.65
RU kinetics			$r_{0P}$	134.31	35
$K_{off}$ $\left(s^{-1}\right)$	100	150	$r_{0N}$ $\left(s^{-1}\right)$	134.31	14
$K_{basic}$ $\left(s^{-1}\right)$	12	150	α (−)	25.184	25.184
			Upscaling		
			$\alpha_{XB}$ (MPa)	22.83e3	4e3

### 2.2 Atrial fibrillation-induced remodeling, *AF-MBS2023*

To incorporate the AF-induced remodeling effect in the MBS2023 model at the basal rate, we adopted an incremental approach, as shown in our previous work (Mazhar et al., 2023). In brief, the first step was to include the electrical remodeling effect induced by the adaptation of ionic currents (Grandi et al., 2011; Chang et al., 2014), followed by the AF-induced increased CaMKII phosphorylation effect (Neef et al., 2010; Heijman et al., 2014b), contractile (Belus et al., 2010; Fakuade et al., 2024), and $Ca^{2+}$ -handling remodeling effects, as shown in Table 2. Among ionic current remodeling, the reduction of $I_{\mathrm{Kur}}$ may occur in a subpopulation of patients with AF as it remains significantly unaffected in many studies (Schotten et al., 2001). Based on this, we have simulated a case where $I_{\mathrm{Kur}}$ reduction is reversed from AF-induced remodeling (as shown in Supplementary Figure S5, case 2, in red dotted line).

**TABLE 2:** Atrial fibrillation-induced remodeling effect on model parameters.

Remodeling	Parameters modified	References
Electrical	$g_{Na}↓$ 10%, $g_{CaL}↓$ 50%, $g_{to}↓$ 80%, $g_{Ks}↑$ 200%, $g_{K1}↑$ 200%, and $k_{NaCa}↑$ 40%	Grandi et al. (2011), Chang et al. (2014)
CaMKII	Expression: CaMK0 ↑ 40%; activation: $alpha_{CaMK}↑$ 200%; $beta_{CaMK}↓$ 50%	Neef et al. (2010), Heijman et al. (2014b)
MCF	$k_{off}↓$ 50%, γ↓ 30%, and [TnC]↓ 25%	Belus et al. (2010), Fakuade et al. (2024)
Sarcoplasmic reticulum	RyRo_k_ RyRc_k_ ↓ 50%, kSRleak ↑ 25%, $k_{4}↓$ 20%, and SERCAKmf ↑ 15%	Manual tunning

Following the incremental approach, we incorporated the myofilament remodeling effect into the model, referred to as mechano-calcium feedback (MCF) remodeling. In accordance with the experiments, the AF impairs the force of contraction (Schotten et al., 2007; 2002; Dobrev and Wehrens, 2017), activation and relaxation rates, and the sensitivity of the thin filament (Belus et al., 2010; Fakuade et al., 2024). The AF-induced sensitization of the Ta–pCa curve is shown in Supplementary Figure S3 (red vs. blue curves). The troponin buffer expression level $\left(TnC_{\max}\right)$ was reduced by 25%, and the XB transition rate (r0) was slowed by 50%. The slowing of XB kinetics was based on an increase in the relative amount of slow myosin heavy-chain β isoform in AF *versus* SR myofibrils, as observed in experiments (Belus et al., 2010). After this, the last AF remodeling effect included was $Ca^{2+}$ -handling remodeling (shown in Table 2), which was achieved by increasing the open probability of RyR channels, as observed in experiments (Dobrev and Wehrens, 2017). For this, we increased the sensitization of RyRs, that is, by reducing the slope $\left(RyRo,c_{k}\right)$ of the activation and inactivation gates. In the supplementary information, we present an AF remodeling case without contractile remodeling mentioned as AF case 1. This shows the impact of contractile remodeling on electrophysiology and $Ca^{2+}$ -handling.

### 2.3 Simulation of $K^{+}$ -channel blockades at basal and higher rates

Having the SR and AF versions of MBS2023, we analyzed the primary and secondary mechanisms induced by the $K^{+}$ -channel block. The pronounced elevation of the AP plateau can activate more $I_{\mathrm{CaL}}$ current and repolarizing current $I_{Kr}$ . Therefore, we simulated the model with the drug while clamping these currents (either $I_{Kr}$ or $I_{\mathrm{CaL}}$ ) to the recorded trace(s) under control conditions, in steady state, and at a basal rate. This simulation was repeated i) using 4-AP and AVE0118, ii) under SR and cAF conditions, and iii) at currents $I_{Kr}$ and $I_{\mathrm{CaL}}$ . At the basal rate, we simulated the block of $K^{+}$ channels with a simple pore block scheme, that is, by varying the maximal conductance of the current using the sigmoidal curve. Using Hill curve formulation, we fixed the concentration for 4-AP to 5 μ M, with a Hill coefficient nH of 1.3 (Wettwer et al., 2004) and a half inhibitory concentration $\left(IC_{50}\right)$ of 8 μ M (Wang et al., 1993) (Supplementary Figure S4 in blue solid line). For simulating the effect of AVE0118 on the model, we used experimental data (Christ et al., 2008) to fit the Hill curve parameters. In this way, for $I_{\mathrm{Kur}}$ , we obtained nH of 0.431 and $IC_{50}$ of 3.126 μ M, and for $I_{to}$ , we obtained nH of 0.385 and $IC_{50}$ of 5.45 mM (Supplementary Figure S4 in red solid and dashed lines, respectively).

We extended the analysis to higher rates for BCL ranging [2 1, 0.5, 0.33, 0.25, 0.2, 0.18]s to evaluate the rate-dependent block behavior of $K^{+}$ -channel blockers. For this, we analyzed electrophysiological characteristics using the biomarker APD and the maximal upstroke velocity $\left(dV/dt_{\max}\right)$ . In addition, we analyzed the inotropic response by computing the maximal peak of active force $\left(Ta_{\max}\right)$ for each BCL.

### 2.4 Analysis of the role of $K^{+}$ -channel blockade on AF-induced burden

#### 2.4.1 $Ca^{2+}$ -dependent EAD (phase 2 EAD) and DAD induction

Triggered activity (TA) is the basis of extrasystole that can lead to the occurrence of tachyarrhythmias and is maintained by reentrant mechanisms. EADs interrupt the repolarization during either phase 2 and phase 3 of the AP, whereas DADs initiate after full repolarization. Conventionally, phase 2 EADs are associated with APD prolongation, resulting from an imbalance between inward and outward sarcolemmal ion currents, and are promoted by bradycardia or pauses. Phase 2 EADs may also be $Ca^{2+}$ -dependent and have some common characteristics with DAD initiation. RyR opening, if it occurs during the resting potential phase, gives rise to DADs; otherwise, it initiates phase 2 EADs when they are present during the early repolarization phase of the AP. One proposed mechanism for AF initiation and maintenance is the TA induced by EADs and DADs in the model. In this work, we analyzed the role of the $K^{+}$ -channel block in reversing the AF-induced burden by elucidating EADs that are promoted by either bradycardia (phase 2 EADs) or tachycardia (phase 3 EADs) and pauses. In addition, we have also analyzed the $K^{+}$ -channel block response toward the DAD induction in the model. For elucidating phase 2 EADs, we reduced the repolarization reserves (RRs) of the cell through i) modulation of $Ca^{2+}$ -handling via $Ca^{2+}$ –AP backward coupling and ii) the AP– $Ca^{2+}$ forward pathway by altering maximal ionic conductance.

At the basal rate, modulation of $Ca^{2+}$ -handling induced phase 2 EADs in the AP. For this, we have reduced the slope of the RyR gate dependent on the ${\left[Ca^{2+}\right]}_{SR}$ content by 10 times. For the forward coupling AP– $Ca^{2+}$ , we varied a combination of maximal conductance(s) and paced the model to BCL = 2s for 70sec. In particular, under SR, we simulated a combination of increased $G_{\mathrm{CaL}}$ (200%–500% of baseline [BL]) and reduced $G_{\mathrm{Kur}}$ (10%–50% of BL). Based on this variation, the model induced four possible abnormalities in the AP: APs with EADs (o), APs with EADs on alternating beats (A-EADs x), APs with repolarization failure (RF open square) (Figure 6), and APs with DADs (Figure 8; Supplementary Figure S11). For a subset of the APs, for instance, a significant increase in $G_{\mathrm{CaL}}$ (6*BL) and $G_{\mathrm{Kur}}$ (BL*0.9), we found RyR-modulated DADs in the model (Supplementary Figure S11). To compare the role of $K^{+}$ -channel blockade on DADs, initiated by a different underlying mechanism, we followed another protocol where the model was rapidly paced at 10 Hz, followed by a pause and back to slower BCL = 2s, as shown in Figure 8.

Under AF conditions, we repeated the same approach, with a different combination of maximal conductance, that is, a 300–600% increase in $G_{\mathrm{CaL}}$ and a reduction of $G_{Ks}$ to 10%–70% of BL. In addition, to have enough $Ca^{2+}$ in the cytosol, we reversed the 50% AF-induced reduction in $G_{\mathrm{CaL}}$ . Using various combinations of RR in the AF cell model, we mapped the AP response into the same three classes of abnormalities, as shown in Figure 6. To assess the role of contraction-related parameters in the vulnerability associated with EADs, we have varied sarcomere length (SL), slope (γ) , and EC50 (kd) of the Ta–pCa curve over a range of [−50% to 50%] under both SR and AF conditions.

#### 2.4.2 Phase 3 EAD induction

Phase 3 EADs form the basis for a premature beat that can re-induce AF (Burashnikov and Antzelevitch, 2003; 2006). Phase 3 EADs can occur either as large depolarizations, as TAs, or with smaller depolarization amplitudes, or even without depolarization, but in the form of slowing of repolarization (Szabo et al., 1994; 1995; Damiano and Rosen, 1984). The takeoff potential of EADs ranges from −45 to −70mV; therefore, there is no role of $I_{\mathrm{CaL}}$ reactivation. The hypothesis formulated by Morotti et al. (2016) was tested in our human atrial model, where reactivation of the nonequilibrium $I_{Na}$ channel induced phase 3 EADs. In addition, in some studies, simultaneous interaction of sympathetic and parasympathetic systems can elicit phase 3 EADs and TA in humans (Bettoni and Zimmermann, 2002).

For phase 3 EAD elucidation, following the experimental protocol (Burashnikov and Antzelevitch, 2003), we first simulated the model at a very fast rate of 10 Hz for 20 s, followed by a pause, and then returned to SR at 1 Hz. To permit the reactivation of the $I_{Na}$ channel, we sped the recovery from inactivation by a factor of 1e^-3^ and shifted the availability curve from 15 mV to the positive potential, as suggested by Rivolta et al. (2002). Apart from this, we made a few modifications to the model: to reflect parasympathetic activation by including the acetylcholine-activated potassium current $I_{\mathrm{KACh}}$ , as formulated by Grandi et al. (2011). In addition, to have a negative AP plateau, we fixed $G_{\mathrm{CaL}}$ = BL*0.8 and $G_{\mathrm{Kur}}$ = BL*3. We repeated the simulation by varying the contraction parameters ( $k_{\mathrm{off}}$ , γ , and $k_{d}$ ) one at a time to evaluate the role of contraction in the reinitiation of AF in the form of phase 3 EADs.

## 3 Results

### 3.1 Characteristics of updated SR and AF versions of MBS2023

Under SR conditions, the AP morphology of the MBS2023 model was modified from the triangular AP with no dome to an AP with a sustained and positive plateau (Supplementary Figure S5, top left panel, in blue solid line). The updated MBS2023 model has a longer APD and a lower upstroke amplitude. Despite all the updates in the model, the key characteristic of the model, that is, the delay in CaT propagation from subspace (*ss*) to the bulk compartment (*bc*), is preserved. The AF version of the model has a triangular AP, with an elevated plateau, a hyperpolarized Resting Membrane Potential (RMP), and a short APD (Supplementary Figure S5 in red solid line). Under AF conditions, $CaT_{bc}$ exhibits an increased diastolic level with an almost unchanged systolic peak compared with $CaT_{bc}$ in SR; this is in line with the experimental data shown by Fakuade et al. (2024) and Voigt et al. (2014). Under AF conditions, the ${\left[Ca^{2+}\right]}_{SR}$ content is preserved (Fig. S5 $J_{bc-SERCA}$ flux red vs. blue). The $Ca^{2+}$ binding affinity to TnC is enhanced because of the increased sensitization of the Ta–pCa curve (Supplementary Figure S3), as observed in experiments (Belus et al., 2010; Fakuade et al., 2024). The Ta developed is depressed and exhibits relatively slower kinetics under AF conditions than under SR conditions (Supplementary Figure S5 Ta panel, red vs. blue), as also observed in experiments (Schotten et al., 2007; 2001; 2002).

Electrophysiological characteristics of AF case 1, that is, without TnC remodeling, are the same as AF condition, whereas the $Ca^{2+}$ –TnC bound is reduced, resulting in reduced Ta (Supplementary Figure S5 in red dashed line). In AF case 2, that is, reversing the AF-induced $I_{\mathrm{Kur}}$ remodeling alone results in a large abbreviation of APD, reduced CaT, and a depressed Ta (Fig. S5 dotted line) because of the loss of the AP-sustained plateau phase. Interestingly, in AF case 2, the $I_{\mathrm{Kur}}$ current peak is not fully restored to that present in SR (blue vs. red dotted line), highlighting the presence of the positive feedback from the sustained plateau of the AP shape in SR.

### 3.2 Positive inotropic response of $K^{+}$ -channel blockers under SR and AF conditions

**FIGURE 2:**
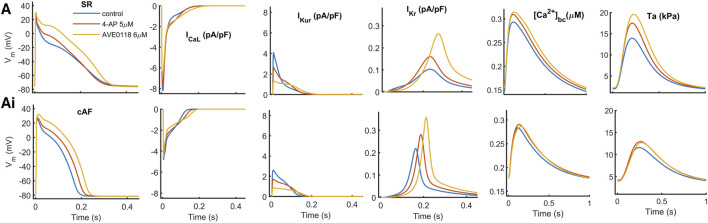
Effects of low-dose concentration of the $K^{+}$ -channel block, 4-AP (5 μ M) (in red) and AVE0118 (6 μ M) (in yellow) on the model. Action potential (AP), ionic current: $I_{\mathrm{CaL}}$ , $I_{\mathrm{Kur}}$ , $I_{Kr}$ , $Ca^{2+}$ -transient in bulk compartment $CaT_{bc}$ , and active tension (Ta) is shown under SR (A: upper row) and AF (Ai: bottom row) conditions.

Under SR conditions, a low-dose concentration of 4-AP (5 μ M) shortens the APD and enhances the plateau phase, as shown in Figure 2A (in red). Because of the elevated plateau potential, the response of the $I_{\mathrm{CaL}}$ channel is biphasic, with a slight decrease in the peak, followed by an enhancement in the plateau (Figure 2A, second panel). This allows more $Ca^{2+}$ to enter the cytosol (Figure 2A fifth panel), which leads to a positive inotropic response of the model (Figure 2A sixth panel). In the presence of AVE0118 (6 μ M), the model shows a similar response under SR, with some differences. AVE0118 lengthens the APD, and the elevation of the plateau potential is more pronounced than that in 4-AP, thus strengthening the positive inotropic response (Figure 2A in yellow).

On the same lines, under AF conditions, both $K^{+}$ -channel blockers enhance the plateau potential; therefore, the positive inotropic response is preserved (Figure 2Ai in red and yellow), with a minor relative change. However, the elevation of the plateau is more pronounced when AVE0118 is applied (Figure 2Ai in yellow), which is not equivalently translated into an increase in Ta compared to 4-AP.

### 3.3 Elucidating the mechanisms of paradoxical APD shortening upon the $K^{+}$ channel block

**FIGURE 3:**
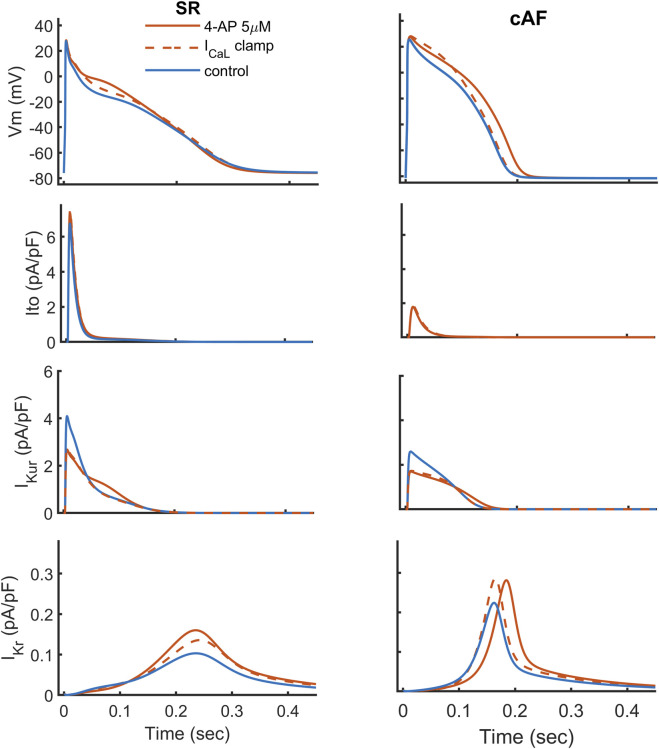
4-AP response on the action potential under $I_{\mathrm{CaL}}$ clamped to a waveform recorded under no-drug conditions for SR (column on left) and cAF (column on right). Ionic currents $I_{to}$ (second row), $I_{\mathrm{Kur}}$ (third row), and $I_{Kr}$ (bottom row) are compared.

**FIGURE 4:**
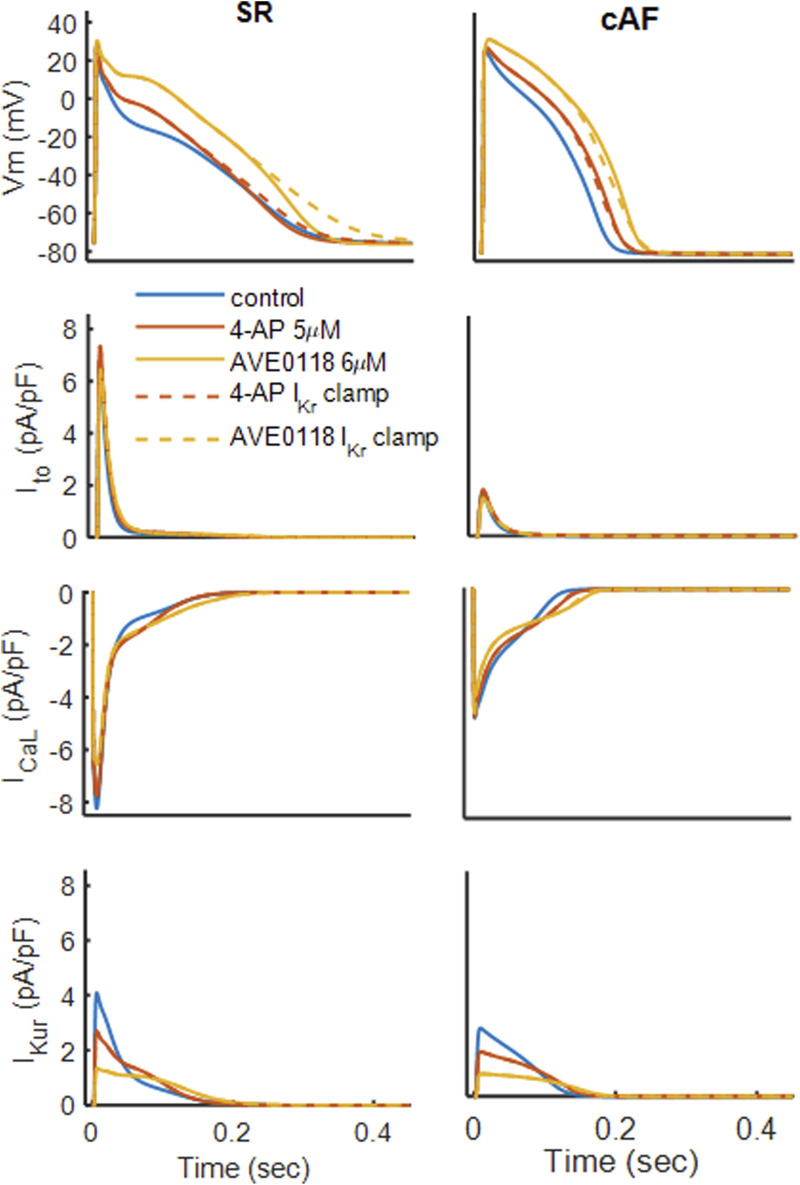
4-AP response on the action potential under $I_{Kr}$ clamped to a waveform recorded under no-drug conditions for SR (column on left) and cAF (column on right). Ionic currents $I_{to}$ (second row), $I_{\mathrm{CaL}}$ (third row), and $I_{\mathrm{Kur}}$ (bottom row) are compared.

The drug-induced elevation of the AP plateau following the $K^{+}$ -channel block leads to a substantial increase in both $I_{\mathrm{CaL}}$ and, subsequently, $I_{Kr}$ . To dissect the causal contribution of these currents to APD changes under drug application, we focused on $I_{\mathrm{CaL}}$ and $I_{Kr}$ under both SR and AF conditions. Using the recorded drug-free $I_{\mathrm{CaL}}$ or $I_{Kr}$ traces, we simulated the model with a $K^{+}$ -channel block under both SR and AF conditions. The corresponding results are shown in Figures 3, 4. Under SR conditions, clamping the $I_{\mathrm{CaL}}$ current attenuated—but did not fully eliminate—the drug-induced elevation of the AP plateau for both drugs. APD remained largely unchanged in the case of 4-AP (Figure 3 in dashed line), whereas the APD-prolonging effect of AVE0118 (Supplementary Figure S7 in dashed line) was fully abolished. For both drugs, the voltage-mediated increase in $I_{Kr}$ was reduced but not completely reversed (Figure 3; Supplementary Figure S7), indicating that this secondary increase in $I_{Kr}$ is driven primarily by sustained depolarization rather than by the increased $I_{\mathrm{CaL}}$ plateau current. Under AF conditions, the $I_{\mathrm{CaL}}$ clamp completely reversed the APD lengthening induced by both drugs (Figure 3; Supplementary Figure S7). However, despite the restoration of APD, the AP plateau paradoxically increased, with a more pronounced effect observed for AVE0118. Under these conditions, the secondary increase in $I_{Kr}$ remained minimally affected, reinforcing our conclusion that the drug-induced increase in $I_{\mathrm{CaL}}$ plays a limited role in augmenting $I_{Kr}$ also in AF.

The effect of clamping $I_{\mathrm{CaL}}$ fully reverses the positive inotropic effect in AF for both drugs (Supplementary Figure S8). Under AF conditions, the delay in ${\left[Ca^{2+}\right]}_{ss}$ decay and ${\left[Ca^{2+}\right]}_{bc}$ peak imposed by AVE0118 because of the slowing repolarization is fully restored (Supplementary Figure S8 inset). Hence, the positive inotropic response induced by the $K^{+}$ -channel blockers is due to the enhanced plateau during phase 2 of the repolarization. Interestingly, with the $I_{\mathrm{CaL}}$ clamp, under AF case 1 condition, the drug-induced increase in $Ca^{2+}$ is still present, resulting in a slight elevation of Ta because of an increase in the Ca–TnC bound concentration. This implies that the increase in Ca–TnC buffering can restore the depression of contractility under AF conditions.

Clamping $I_{Kr}$ converts the 4-AP-induced APD shortening into APD prolongation under SR conditions, without altering the plateau potential (Figure 4) for both drugs. This indicates that the 4-AP-mediated elevation of the plateau potential enhances $I_{Kr}$ activation, thereby shortening APD. With AVE0118, clamping $I_{Kr}$ further accentuates APD prolongation, confirming that AVE0118-induced APD lengthening arises primarily from increased $I_{\mathrm{CaL}}$ (as shown in Figure 3) rather than augmented $I_{Kr}$ activation. As expected, clamping $I_{Kr}$ produces minimal effects on $Ca^{2+}$ handling and thus on contractility.

### 3.4 $K^{+}$ -channel block-induced positive inotropic response is retained under cAF conditions

**FIGURE 5:**
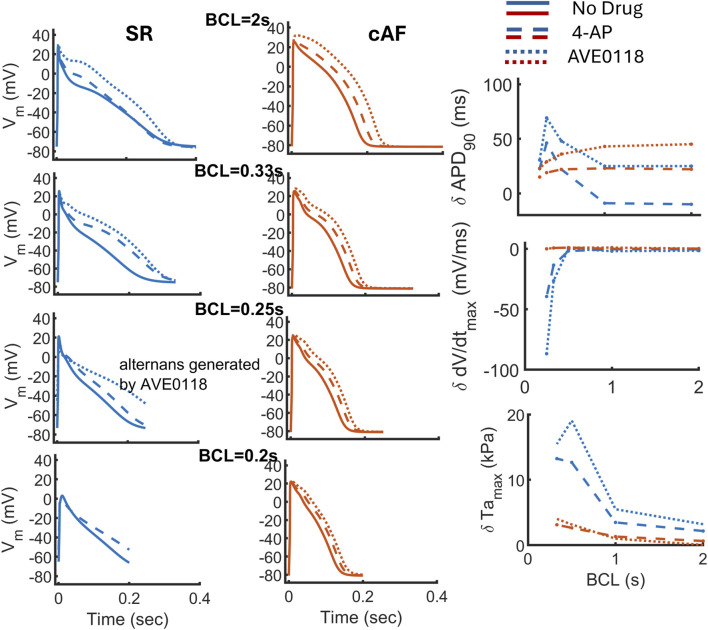
Rate-dependent response of $K^{+}$ -channel blockers 4-AP (in dashed line) and AVE0118 (in dotted line) under SR (left column) and AF (right column) conditions. The simulation results are shown for BCL 2, 0.33, 0.25, and 0.2 s. The biomarkers used for analysis are the change in APD δ APD and change in the maximal upstroke velocity $\delta dV/dt_{\max}$ , where δ is the difference in the biomarker value obtained under drug conditions with respect to the control.

The rate dependency of the $K^{+}$ -channel block was assessed by extending the analysis to higher Basic Cycle Length (BCL) rates, ranging from 2 to 0.2 s, as shown in Figure 5. The rate-dependent shortening of APD is preserved in the updated MBS2023 model under both SR and AF conditions, with the slope flattened under SR conditions but remaining unchanged under AF conditions (not shown). Under SR conditions, the APD shortening effect of 4-AP at BCL = 2 and 1 s is converted into significant prolongation of BCL from 0.5 s onward. This change in APD is quantified using δ APD for a biomarker B, δ = $B_{\mathrm{drug}}$ − $B_{\mathrm{control}}$ , where δ APD is negative for slow rates (BCL = 2 and 1s) and becomes positive from BCL = 0.5 s onward (Figure 5). In contrast, the response of AVE0118 on δ APD is always positive, that is, lengthens the APD for all BCL. Under SR conditions, the AP upstroke is least affected at lower BCLs and slows down for higher rates, from BCL = 0.33 s and onward, as shown by the change in the maximal upstroke velocity $\delta dV/dt_{\max}$ . This effect is strengthened in the case of AVE0118 (in dashed blue line); therefore, it can be related to the profound lengthening of the APD at higher rates, from BCL = 0.33 s onward. The prolonged APD does not have enough time to complete at higher rates, resulting in depolarized RMP, thus lowering the availability of $Na^{+}$ channels, as shown in Figure 5 (on the left panel from BCL = 0.33 s onward). Under cAF conditions, the response of δ APD is always positive, that is, lengthening for all BCLs using both drugs (Figure 5, right panel in red). During cAF, the APD is relatively short; therefore, the drug-induced lengthening is not enough to produce any change in $\delta dV/dt_{\max}$ using either of the drugs, as shown in Figure 5 (right panel in red). The drug-induced positive inotropic response, as quantified by $\delta Ta_{\max}$ , is maintained at all rates under both SR and AF conditions ( $\delta Ta_{\max}$ always positive in Figure 5, bottom right panel). This positive inotropic response is enhanced at higher rates, particularly under SR conditions (Figure 5, bottom right panel, blue curves).

### 3.5 $K^{+}$ -channel block can exacerbate the phase 2 EAD-induced proarrhythmicity

**FIGURE 6:**
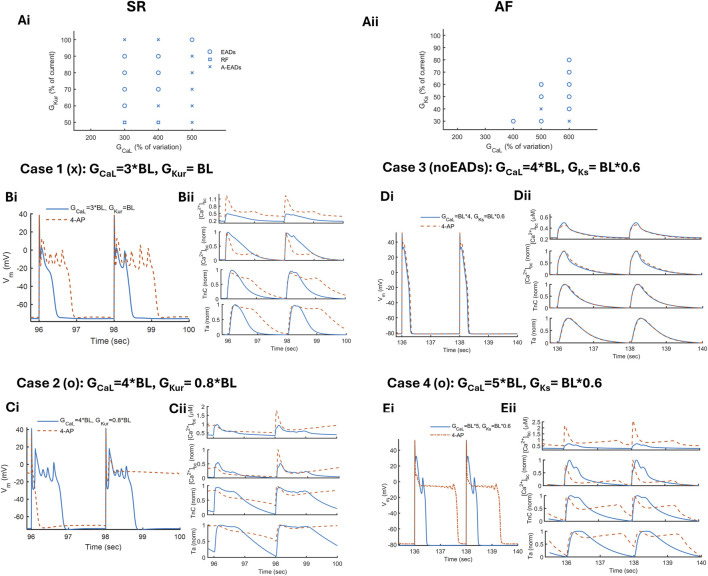
4-AP response on phase 2-EADs under SR and cAF conditions. By pacing at a slow rate of BCL = 2s, for 70 beats, the model elucidated three different forms of abnormalities: APs with EADs (o), APs with EADs on alternating beats (A-EADs x), and APs with repolarization failure (RF open square) obtained by the combination of varying maximal conductance, for instance, for SR, (Ai) $G_{\mathrm{CaL}}$ 2–5 times and $G_{\mathrm{Kur}}$ 100%–50% and, for AF, (Aii) $G_{\mathrm{CaL}}$ 2–6 times and $G_{Ks}$ 100%–30% of the baseline (BL) value. The response for 4-AP (in red dashed line) is analyzed under SR **(B)** case 1 A-EADs and **(C)** case 2 EADs, along with under AF **(D)** case 3 with no EADs and **(E)** case 4 EADs.

We analyzed the effects of 4-AP on the inducibility and vulnerability of phase 2 EADs, as well as on AP and contractility, under both SR and AF conditions, as shown in Figure 6. The outcome of the maximal conductance variation map is in the form of three different abnormalities, namely, EADs (o), A-EADs (x), and RF (open squares), as shown in Figure 6. Under SR conditions, the threshold of inducing EADs is reached by increasing $G_{\mathrm{CaL}}$ up to 3*BL, and afterward, the reduction in $G_{\mathrm{Kur}}$ intensifies it (Figure 6Ai). In contrast, under cAF conditions, this threshold is shifted to a higher $G_{\mathrm{CaL}}$ value and is induced by the combination of $G_{\mathrm{CaL}}$ 4*BL+ $G_{Ks}$ 0.3*BL (Figure 6Aii). This can be related to hastened repolarization during cAF, where $I_{\mathrm{CaL}}$ is also reduced, impairing the conditions to induce EADs.

To study the response of 4-AP under SR, we have considered two cases from the map in Figures 6Ai, that is, case 1 with A-EADs (Figure 6B) and case 2 with EADs (Figure 6C). Case 1 elicits AP with A-EADs, where RR is reduced by increasing $G_{\mathrm{CaL}}$ to 3 × BL levels. Two successive APs are shown in Figure 6BiL: the first beat (b1) with slow repolarization and the second beat (b2) with an EAD during phase 2. For both beats, the AP demonstrates a negative notch during the initial phase of the plateau, followed by a depolarization. This is due to the increase in the inward $I_{\mathrm{NaCa}}$ current because of the increased $Ca_{2+}$ entering through the enhanced $I_{\mathrm{CaL}}$ current. For b2 at t = 98.3 s, the AP undergoes another notch, with a very negative value that results in the slowing of the voltage-dependent inactivation (VDI) of the $I_{\mathrm{CaL}}$ current, thus leading to the reactivation of the current. In the bulk cytosolic compartment, the increase in ${\left[Ca^{2+}\right]}_{bc}$ results in increased binding time and concentration of $Ca^{2+}$ –TnC bound and eventually leads to a higher Ta with a slow relaxation (duration lasting for 1.1s) (Figure 6Bii in blue). On the same lines, during b2, the AP depolarization during phase 2 results in multiple peaks of ${\left[Ca^{2+}\right]}_{bc}$ and rebinding of $Ca^{2+}$ –TnC (Figure 6Bii).

In the presence of 4-AP, A-EADs convert into EADs with multiple depolarizations during phase 2 and with larger amplitude, exacerbating the AP oscillations (Bi in red dashed line). The RMP gets depolarized by 3̃mV, resulting in the slowing of $dV/dt_{\max}$ due to lower availability of $Na^{+}$ channels and eventually leading to lower excitability. Meanwhile, a longer AP plateau induced by 4-AP increases the $Ca^{2+}$ level in the cytosol even more, with an initial faster decay followed by a slight slowing. This may be because of the biphasic response of the $I_{\mathrm{CaL}}$ current when 4-AP is applied, that is, it gets reduced during the peak, followed by the slow VDI effect. A large $CaT_{bc}$ allows an increased $Ca^{2+}$ –TnC bound and worsens the Ta relaxation delay (Figure 6Bii, bottom panel). On reducing the RR further, as shown by case 2, induced by a combination of $G_{\mathrm{CaL}}$ 4*BL and $G_{\mathrm{Kur}}$ 0.8*BL, the model demonstrates EADs with slightly alternating APD among b1 and b2 (Figure 6Ci). During the longer APD b1, $Ca^{2+}$ remains bound to the TnC buffer for a longer duration because of the significant elevation in the ${\left[Ca^{2+}\right]}_{bc}$ diastolic level (0.48 μ M), resulting in an increased resting Ta level. In the presence of 4-AP, EADs convert into the AP beat with RF, followed by an AP with a lower upstroke and largely depolarized RMP. During the AP with RF b2, $CaT_{\mathrm{peak}}$ significantly increases, followed by a gradual increase in the diastolic level (Figure 6C). This keeps $Ca^{2+}$ –TnC bound to the peak level even during diastole, and increased binding results in a Ta that never relaxes.

The inducibility of EADs on applying 4-AP is assessed under AF conditions in case 3, with no EADs for $G_{\mathrm{CaL}}$ 4*BL and $G_{Ks}$ BL*0.6 (Figure 6D). When 4-AP is applied, it does not prolong the AP much, with no prominent change in contraction-related traces (Dii). This is possibly because the cAF-induced abbreviated AP with a positive plateau does not allow the $I_{\mathrm{CaL}}$ gate to reactivate, that is, fast recovery from inactivation. Case 4 indicates the exacerbation of EADs induced by 4-AP on AP and contractility, similar to case 1 under SR conditions, but with a large diastolic elevation of ${\left[Ca^{2+}\right]}_{bc}$ . Thus, under both SR and AF conditions, 4-AP can exacerbate the vulnerability of phase 2 EADs by slowing the repolarization and increasing the activation of the myofilaments, with some anti-AF action in the form of reduced AP excitability.

EADs induced by $Ca^{2+}$ –AP backward coupling modulation are completely abolished by 4-AP (Supplementary Figure S15) under both SR and AF conditions. RyR sensitization increases cytosolic $Ca^{2+}$ release, thereby increasing the CDI effect of $I_{\mathrm{CaL}}$ and lowering the AP plateau. A negative plateau slows down the VDI effect (f gate shown in S15) of $I_{\mathrm{CaL}}$ , thus resulting in reactivation of the current and eliciting an EAD. In the presence of 4-AP, the elevation of the AP plateau speeds up the f gate, hence eliminating the EAD.

#### 3.5.1 Desensitization of myofilament can reduce phase 2 EADs

**FIGURE 7:**
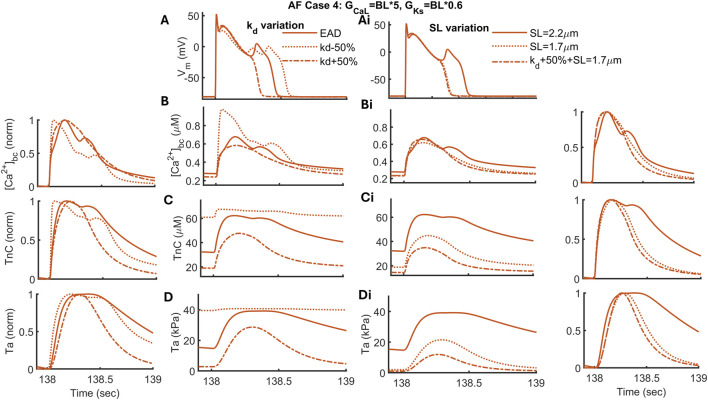
Variation of sarcomere bound to troponin (TnC) sensitivity (kd) (column 2) and length (SL) (column 3) for case 4 under AF, where EAD was elicited by increasing $G_{\mathrm{CaL}}$ 5*BL and $G_{Ks}$ 0.6*BL. Here, the sensitivity of the $Ca^{2+}$ –TnC bound is the amount of $Ca^{2+}$ to produce half of the activated force. kd varied from −50 to +50%, and SL was shortened from 2.2 to 1.7 μ m. A combination of desensitized and shortened sarcomere was assessed (in dot dashed line) to observe its impact on EADs.

EAD induction in the model results in increased activation of the TnC buffer, which gets further exacerbated in the presence of the $K^{+}$ -channel block. In a bidirectional fully coupled model, $Ca^{2+}$ handling can strongly influence the membrane voltage; therefore, we assessed whether reducing the activation time of the TnC buffer can be a practical therapeutic approach for eliminating EADs. Based on this, we varied the thin filament sensitivity in a range of ± 50% of the BL to determine the effects on reduced RR-induced EADs (Figure 7 columns 2 and 3). This was simulated by varying $k_{d}$ of $Ca^{2+}$ bound to TnC, that is, the value of $Ca^{2+}$ , where Ta reaches half its maximal level. Under AF conditions (case 4 of Figure 6), the model demonstrates that a desensitized sarcomere (kd + 50%) can abolish EAD induction in the model (Figure 7A in dot-dashed line). A 50% shift of the Ta–pCa curve to the right has reduced the on-time duration of the TnC buffer (Figure 7C in dot-dashed line and the normalized TnC curve on the left), hence reducing the $Ca^{2+}$ –TnC bound concentration, which lowers the free $Ca^{2+}$ level peak and diastolic value in the cytosol (Figure 7B). A relatively lower $Ca^{2+}$ level eventually reduces the inward mode of $I_{\mathrm{NaCa}}$ , which can make the early repolarization of the AP faster, hence eliminating the EADs (Figure 7A).

In cAF myofibrils, length dependence of activation (LDA) is reduced because of reduced passive myofibril stiffness (Belus et al., 2010). This relates to the upregulation of N2BA at the expense of N2B titin isoform (Belus et al., 2010). Following this finding, we simulated SL variation from 2.2 to 1.7 μ m to determine its impact on the AP with EADs under AF conditions, as shown in Figure 7 (columns 3 and 4). Interestingly, the shorter SL reversed the EAD inducibility in the model (in a dotted line) because of the reduction in the LDA effect, that is, a reduced $Ca^{2+}$ –TnC binding affinity. Consequently, combining $k_{d}$ +50% with shorter SL showed similar results. Overall, desensitization of myofilaments abolishes the EADs in the model by reducing the activation time of the thin filament binding.

### 3.6 $K^{+}$ -channel block can reduce the $Ca^{2+}$ -induced DAD susceptibility

Following the same approach, DADs were induced in a subset of APs exhibiting maximal conductance variation. One such case under SR shows a coexistence of EAD and DADs, as shown in Supplementary Figure S11, where a combined increase in $G_{\mathrm{CaL}}$ by 6 times and a reduction in $G_{\mathrm{Kur}}$ by 0.9 induced strong EADs in phase2, followed by small depolarization in the resting phase (Fig. S11Ai pointed out by arrows). A highly negative notch in the AP results in the slowing of $I_{\mathrm{CaL}}$ inactivation, followed by a reopening; therefore, the appearance of EADs led to further loading of the cell with $Ca^{2+}$ (resting level from 0.14 to 0.5 μ M) and $Na^{+}$ (11 mM). This elevated $Ca^{2+}$ load, when sensed by RyRs, accelerates RyR recovery, thereby increasing spontaneous opening (Supplementary Figure S11). This is also evident from the RyR inactivation gating variable css (Supplementary Figure S11E), which oscillates and has a large recovery rate. A large $J_{\mathrm{rel}}$ increases $J_{up}$ from the cytosol to SERCA, whereas the increased ${\left[Ca^{2+}\right]}_{SR}$ loading expedites $J_{up}$ decay, thus accelerating the overall uptake. To understand the cause–effect relation underlying DAD induction in the model, we evaluated the protocol by fixing ${\left[Ca^{2+}\right]}_{ss}$ to its resting level under control conditions and running the protocol without the drug condition (not shown). The DADs were eliminated by removing the cytosolic $Ca^{2+}$ overload. In the presence of 4-AP, the elevation of the AP plateau bypasses the notch potential, resulting in faster $I_{\mathrm{CaL}}$ inactivation and eliminating the probability of reopening. Consequently, the DADs are eliminated due to reduced ${\left[Ca^{2+}\right]}_{ss}$ , resulting in a decreased inward $I_{\mathrm{NaCa}}$ current (Supplementary Figure S11F in red).

**FIGURE 8:**
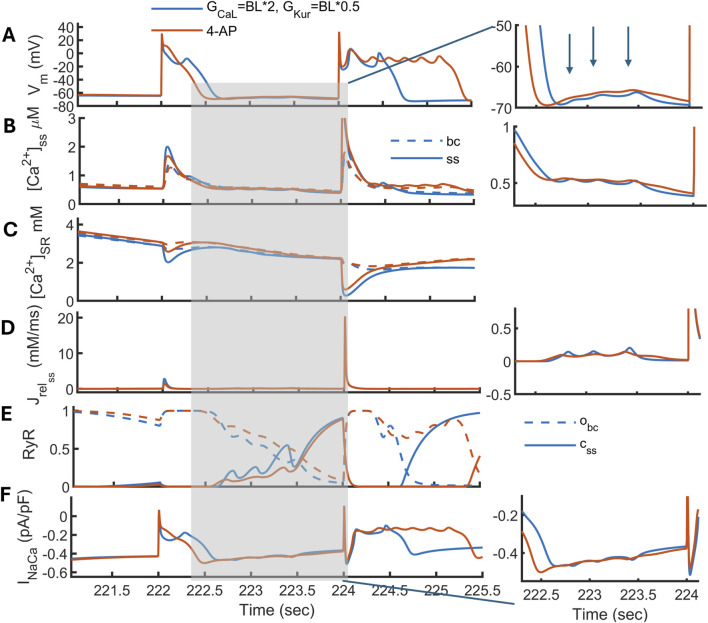
Development of delayed afterdepolarizations (DADs) in the updated MBS2023 model (in blue) under a condition with $G_{\mathrm{CaL}}$ increased by 2 times of the baseline (BL) value and $G_{\mathrm{Kur}}$ reduced by BL*0.5, along with its response in the presence of 4-AP (in red). DAD induction using rapid pacing at 10 Hz, a pause, and a switch back to a slower rate BCL = 2s. **(A)** The action potential Vm, **(B)** $Ca^{2+}$ -transient in the subspace ${\left[Ca^{2+}\right]}_{ss}$ and ${\left[Ca^{2+}\right]}^{bc}$ in dashed line, **(C)** ${\left[Ca^{2+}\right]}_{SR}$ in ss (in solid line) and in *bc* (in dashed line) compartments, **(D)** release flux $Jrel_{ss}$ , **(E)**, RyR activation in bc $\left(o_{bc}\right)$ (in dotted line) and inactivation in ss $\left(c_{ss}\right)$ (in solid line), **(F)** $I_{\mathrm{NaCa}}$ current. The panels are zoomed in time from 222.4 to 224.1 s in the inset from **(A)** to **(F)**.

In the model, the rapid-pacing-induced DADs appeared only in the time frame of 222.4–224 s and disappeared in later APs, as shown in Figure 8. The rapid pacing overloads $Na^{+}$ (from 7 to 9.5 mM) and $Ca^{2+}$ (resting ${\left[Ca^{2+}\right]}_{ss}$ level from 0.14 to 0.54 μ M) in the cytosol, along with ${\left[Ca^{2+}\right]}_{SR}$ from 0.6 to 3 mM. The rapid pacing also increases CaMKII activity, which can contribute to DAD inducibility by increasing RyR phosphorylation and ${\left[Ca^{2+}\right]}_{SR}$ leak. However, fast pacing hinders the release because there is less time for RyRs to recover from inactivation. To confirm the underlying mechanism for DADs, we repeated the protocol by fixing ${\left[Ca^{2+}\right]}_{SR}$ at its resting level, which eliminated DADs, thereby confirming the role of ${\left[Ca^{2+}\right]}_{SR}$ load (not shown). Therefore, a $Ca_{2+}$ -loaded reticulum with a moderate level of $Ca_{ss}$ leads to the depolarization of AP *via* a large $I_{\mathrm{NaCa}}$ current (Figure 8F) that gives rise to DADs.

When 4-AP is applied, the model demonstrates an elevation of the plateau potential, a depolarization of RMP, and a shortened APD (Figure 8A). 4-AP decimates but does not fully eliminate the appearance of small DADs in the model. A reduced $Ca^{2+}$ in the cytosol decreases the inward $I_{\mathrm{NaCa}}$ current, thus reducing the oscillatory behavior in the resting phase. Moreover, a slight increase in the ${\left[Ca^{2+}\right]}_{SR}$ load accelerates $J_{up}$ , which speeds up the CaT decay. For the subsequent beats, 4-AP exacerbates the vulnerability induced by phase 2 EADs, as shown previously in Section 3.5. Overall, DADs induced by cytosolic $Ca^{2+}$ loading (first case) are fully eliminated by 4-AP than those induced by ${\left[Ca^{2+}\right]}_{SR}$ load (second case).

### 3.7 $K^{+}$ -channel block can be effective in preventing phase-3 EADs

**FIGURE 9:**
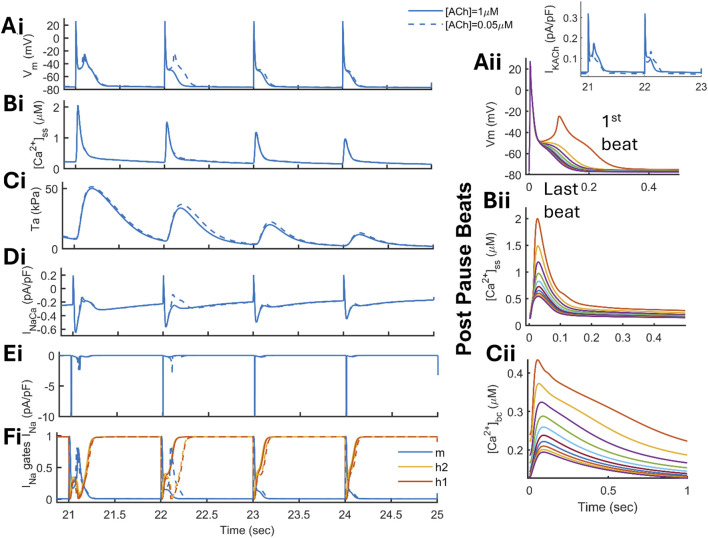
Elucidation of phase-3 EADs in the human atrial electromechanical model. The model was rapidly paced at 10 Hz for 20 s and returned to the sinus rhythm after a pause in the presence of $I_{\mathrm{KACh}}$ with [ACh] of 1 μ M (in solid line) and 0.05 μ M (in dashed line). The transient increase in $Ca^{2+}$ in the cytosol $\left(Ca_{ss}\right)$ (Bi) increases the inward mode of the $I_{\mathrm{NaCa}}$ current (Di). A parallel increase in bulk $Ca^{2+}$ results in transient hypercontractility (Ci). A strong $I_{\mathrm{NaCa}}$ creates a transient prolongation of APD (Ai) and facilitates the reactivation of the $I_{Na}$ current (Ei and Fi). Post-pause beats, when superimposed (on the right), show that a beat-to-beat reduction in ${\left[Ca^{2+}\right]}_{SR}$ loading reverses APD prolongation (Aii) and $Ca^{2+}$ accumulation (Bii and Cii).

Following the rapid pacing for 20 s, the model elicited phase-3 EADs, as shown in Figure 9 (solid line). Under $I_{\mathrm{KACh}}$ (1 μ M)-induced abbreviated APD conditions, the model exhibited a small depolarization during phase 3 (Figure 9Ai) upon returning to SR condition (1 Hz) from rapid pacing (10 Hz). The rapid pacing-induced $Ca^{2+}$ -loading (Figure 9Bi) enhanced the inward $I_{\mathrm{NaCa}}$ current (Figure 9Di). This transient increase in $Ca^{2+}$ in the cytosol resulted in a transient period of hypercontractility (Figure 9Ci), also shown by the post-pause beats superimposed on each other (Figure 9Cii). Thus, the abbreviation of the APD (by $I_{\mathrm{KACh}}$ ) allows strong recruitment of $I_{\mathrm{NaCa}}$ in the development of phase 3-EADs that is responsible for the transient prolongation of APD, hence providing room for the reactivation of $I_{Na}$ channels, as shown in Figures 9Ei,Fi. The take-off potential for the EAD is −48mV, eliminating the role of $I_{\mathrm{CaL}}$ reactivation. In addition, we simulated blocking of the $I_{\mathrm{CaL}}$ current only after the take-off potential time is reached, that is, at t = 21.044 s, and we found a slight shortening of APD (Supplementary Figure S10 in dashed vs. solid line) with no change in the EAD amplitude, confirming a negligible role of $I_{\mathrm{CaL}}$ reactivation. Beat-to-beat transition reduces the ${\left[Ca^{2+}\right]}_{SR}$ load, reducing $Ca^{2+}$ levels in the cytosol; hence, reduced $I_{\mathrm{NaCa}}$ shortens the APD gradually (Figure 9Aii). Reducing the concentration of acetylcholine [ACh] from 1 μ M to 0.05 μ M (Figure 9 in dashed line) or the current amplitude by BL*0.5 (Supplementary Figure S12) can increase the propensity and induction of phase 3-EADs, where the EADs were strengthened in the subsequent post-pause beats. A large $I_{\mathrm{KACh}}$ value hyperpolarizes the AP and modulates the repolarization rate, thereby reducing $I_{Na}$ availability. Therefore, the development of phase 3-EADs is strictly dependent on the ACh-induced abbreviation of the APD, as observed in experiments based on the whole canine right atrium (Burashnikov and Antzelevitch, 2003).

**FIGURE 10:**
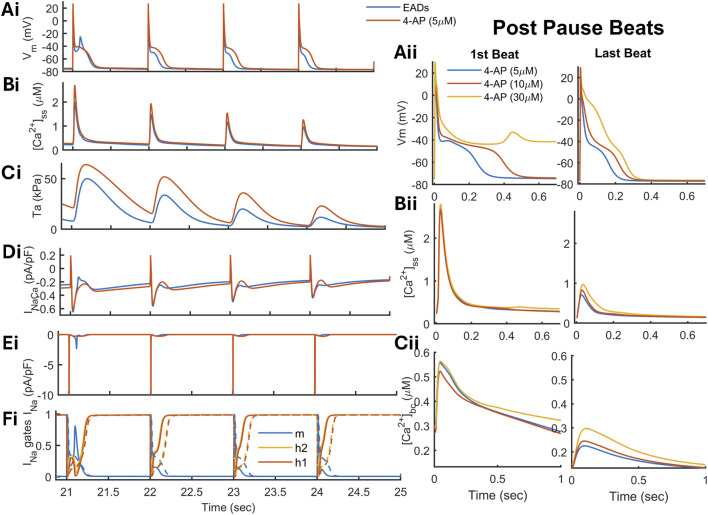
$K^{+}$ -channel block has an antiarrhythmic response on phase-3 EADs. 4-AP (5 μ M), when applied to the model, eliminates the EADs by elevating the plateau potential of the AP (Ai) and a slower repolarization phase that reduces the probability of $I_{Na}$ reactivation (Ei and Fi). An increased $Ca^{2+}$ level in the cytosol (Bi), the positive inotropic response of 4-AP (Ci), and an increased $I_{\mathrm{NaCa}}$ inward current (Di). The right panel shows a comparison of transient (on the left) and steady response (on the right) of 4-AP on Vm (Aii), $Ca^{2+}$ in subspace (Bii), and $Ca^{2+}$ in bulk (Cii).

When 4-AP (5 μ M) is applied to the model, it eliminates phase 3-EADs, as shown in Figure 10. Blocking of $I_{\mathrm{Kur}}$ results in a positive plateau potential of 8 mV higher than the required take-off potential (Figure 10Ai in red). The sustained plateau slows down the repolarization, restricting the reopening of the $I_{Na}$ channel (Figures 10Ei,Fi). The slow repolarization also results in a transient depolarization of RMP (by 1 mV), resulting in a reduced availability of Na+ channels. The response of 4-AP on the AP and contractility is dose-dependent (Figure 10 right panels), where for lower-dose concentrations of 4-AP (5 μ M in blue and 10 μ M in red), the transient response of 4-AP was the elimination of EADs, with an enhanced plateau and a large prolongation of APD (Figure 10Aii). In contrast, for a higher dose of 4-AP (30 μ M), there is a transient phase of RF (Figure 10Aii in yellow) that was abolished when a steady state was reached (on the right). In the transient phase, the dose-dependent positive inotropic response was disrupted (Figure 10Cii on the left) and was restored in a steady state (on the right). The same simulation is repeated using AVE0118 (6 μ M), resulting in transient RF and an APD prolongation in the steady state more pronounced than 4-AP (Supplementary Figure S13). By varying the myofilament contraction-related parameters, the model demonstrated that sensitization of the thin filament (by 50%) can increase the inducibility of phase 3 EADs (Supplementary Figure S14, dotted line) due to increased thin filament activation. A shorter sarcomere (SL = 1.7 μ m) can reduce hypercontractility during the post-pause transient phase and slow repolarization, thereby reducing the probability of $Na^{+}$ channel reopening.

## 4 Discussion

### 4.1 Characteristics of the updated MBS2023 and MBS2023-AF models

In this work, we have assessed the anti-arrhythmogenic potential of the $K^{+}$ -channel block using low concentrations of $I_{\mathrm{Kur}}$ -specific and nonspecific drugs under SR and AF conditions. We have updated the SR and developed an AF version of our previous human atrial electromechanical cardiomyocyte model, MBS2023 (Mazhar et al., 2024). Contractile remodeling is the hallmark of AF-associated vulnerability effects (Allessie et al., 2002), which remains for several weeks after the cardioversion, contributing to the high risk of atrial thrombus formation and stroke risk (Manning et al., 1994). Therefore, in the AF version, the model includes, in addition to conventional electrophysiological remodeling effects, the experimentally informed impaired MCF effect. In the model, the AF-induced $Ca^{2+}$ -handling remodeling effect was due to RyR channel dysfunction, leading to increased channel opening. The underlying molecular substrate for RyR dysfunction can be related to three different mechanisms: enhanced ${\left[Ca^{2+}\right]}_{SR}$ loading because of increased SERCA activity (Voigt et al., 2014) in paroxysmal AF, reduced levels of the inhibitory microRNA-106b-25 cluster (Chiang et al., 2014), or the reduced RyR-stabilizing protein JPH2 (Beavers et al., 2013).

In the AF model, increased RyR channel activity resulted in increased $J_{\mathrm{relss}}$ flux, producing $CaT_{ss}$ that increases earlier and decays more rapidly (Supplementary Figure S8 top row SR vs. cAF). During the latter phase of $CaT_{ss}$ , decay was further accelerated because of increased $Ca^{2+}$ efflux through the $I_{\mathrm{NaCa}}$ channel. The reduced systolic $CaT_{ss}$ level led to a decrease in cytosolic $CaT_{bc}$ *via* decreased diffusion $J_{\mathrm{diff}}$ from *ss* to *bc*. In the model, the elevated CaT diastolic level in AF compared to SR was due to the AF-induced increase in CaMKII phosphorylation activation, as observed in experiments (Neef et al., 2010). Based on this, an experimentally verified CaT under AF remodeling conditions maintains the positive inotropic response to $K^{+}$ -channel block, as observed in human atrial trabeculae (Schotten et al., 2007).

**FIGURE 11:**
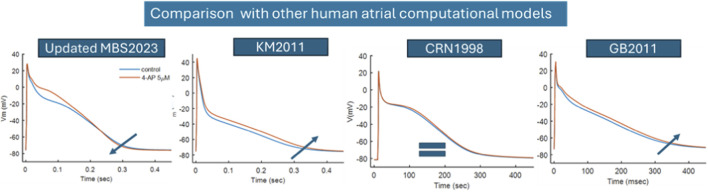
Comparison of the 4-AP (5 μ M) response using four different human atrial computational models. From left to right: updated MBS2023 by Mazhar et al. (2024), Koivumaki2011 (KM2011) by Koivumäki et al. (2011), Courtemanche1998 by Courtemanche et al. (1998), and Grandi2011 (GB2011) by Grandi et al. (2011).

In the presence of 4-AP (5 μ M), at the basal rate, the updated SR model demonstrates shortening of the APD, in line with experimental results (Wettwer et al., 2004; Ford et al., 2016). Our model shows the correct $I_{\mathrm{Kur}}$ block response, which is lacking in existing human atrial action potential models [Koivumaki2011 (KM2011) (Koivumäki et al., 2011), CRN1998 (Courtemanche et al., 1998), and GB2011 (Grandi et al., 2011), as shown in Figure 11]. Both KM2011 and GB2011 have a short APD with a triangular AP shape (type-3 morphology), whereas CRN1998 has a spike-and-dome AP shape (type-2 morphology). Across various atrial models, the 4-AP drug response lengthens the APD in type-3 morphology, whereas in type-2 morphology, there is no change in the APD, as shown in Figure 11. This is possibly because of the sustained AP plateau observed in the updated MBS2023 and CRN1998 models. When 4-AP is applied, the elevation of the plateau phase activates more $I_{Kr}$ current, and this effect is less pronounced in the CRN1998 model.

The functional effects of 4-AP in multicellular or tissue experiments, compared to other $I_{\mathrm{Kur}}$ -specific and high-affinity drugs such as XEN-D0103 and MK-0448 ( $IC_{50}$ in nM), are small. Therefore, a higher dose of 4-AP is needed to achieve the same level of selective Kv1.5 inhibition observed with other selective small molecules in an isolated-channel assay.

### 4.2 $K^{+}$ -channel block can exacerbate the phase 2 EAD-induced proarrhythmicity

Phase 2 EADs promote arrhythmias by either triggering TAs or causing electrical heterogeneity that is liable to unidirectional conduction block and reentrant circuit maintenance (Weiss et al., 2010). EADs can also be involved in the generation and maintenance of AF (Heijman et al., 2014a). In the model, the EADs were induced by increasing the inward flow of $Ca^{2+}$ in the cytosol, which enhanced the forward mode of the $I_{\mathrm{NaCa}}$ current and formed a negative notch. A more negative notch during the plateau strengthens the EAD amplitude and frequency. This is because a negative notch can slow down the VDI effect and favor the reopening of the $I_{\mathrm{CaL}}$ channel. Consequently, the application of the drug exacerbates this oscillatory behavior by converting an AP with A-EAD to EAD and EAD to RF (Figure 6C). Interestingly, using the dynamic clamp approach, Kettlewell et al. found a similar potential relevance of $K^{+}$ current reduction or inhibition to AF in human and rabbit atrial cardiomyocytes (Kettlewell et al., 2019). Moreover, based on computational modeling, Heitmann et al. (2021) found that the cells with bistable membrane dynamics (liable to RF) can initiate fibrillation if they remain depolarized beyond the refractory period of their neighboring cells.

In experiments, Kv1.5 loss-of-function channelopathy is a risk factor for repolarization deficiency and AF (Olson et al., 2006). Therefore, the worsening of repolarization instability toward RF upon $K^{+}$ -channel blockade could increase the arrhythmic risk and susceptibility to AF.

### 4.3 Desensitization of myofilaments can abolish phase 2 EADs

A slight change in myofilament $Ca^{2+}$ sensitivity may result in significant changes in CaT as $Ca^{2+}$ –TnC binding is the most considerable component of dynamic $Ca^{2+}$ buffering during the cardiac cycle (Smith and Eisner, 2019). This, in turn, may lead to AP remodeling and perturbed intracellular $Ca^{2+}$ handling, creating both an arrhythmogenic substrate and a trigger in the atrial myocardium. Accordingly, we tested the hypothesis whether reducing the activation time of the TnC filament would decrease the $Ca^{2+}$ load in the cell, thereby eliminating EADs. We found that lowering the on-time of TnC buffer by reducing the sensitivity of $Ca^{2+}$ –TnC buffer can eliminate the EADs under AF conditions (Figure 7) but not under SR conditions (Supplementary Figure S9). Myofilaments with AF exhibit higher $Ca^{2+}$ sensitivity, which is associated with increased phosphorylation of ALC-2 (atrial isoform of myosin light chain) and desmin (Belus et al., 2010; Kockskämper et al., 2008). Sensitization of the myofilament under AF conditions may contribute to electrical remodeling and the self-perpetuation of atrial arrhythmias. In an isolated mouse heart, myofilament $Ca^{2+}$ sensitization (by EMD 57033 or by troponin T mutation) was reported to induce arrhythmia by shortening of the ERP, APD, slowing of CV, predisposing to EADs, and TA (Baudenbacher et al., 2008). In Langendorff-perfused mouse hearts, the desensitization of myofilament to $Ca^{2+}$ using blebbistatin has no impact on atrial arrhythmia inducibility and episode duration for the physiological value of ${\left[K^{+}\right]}_{o}$ (5.4 mM) (Fakuade et al., 2024). However, in some studies, a reduction in $Ca^{2+}$ –TnC buffering (as a result of desensitization of TnC) is associated with increased $Ca^{2+}$ sparks and with the propagation of intercellular arrhythmogenic $Ca^{2+}$ waves (Fakuade et al., 2024). In this study, we do not consider the spatial resolution of dyadic space; therefore, we cannot demonstrate $Ca^{2+}$ sparks or their intracellular propagation as $Ca^{2+}$ waves, as mentioned in the Limitation Section.

### 4.4 $K^{+}$ -channel block can decimate the $Ca^{2+}$ -induced DAD susceptibility

$Ca^{2+}$ mediates the cardiac excitation–contraction coupling, where an excitation of AP leads to a $Ca^{2+}$ level that mediates mechanical contraction and relaxation. Due to the strong coupling between $Ca^{2+}$ and AP, $Ca^{2+}$ -induced abnormalities can lead to arrhythmia initiation and maintenance. $Ca^{2+}$ -induced DADs promote AF maintenance by contributing to the electrical and structural remodeling in the atria (Nattel and Dobrev, 2012). Based on this, $Ca^{2+}$ -handling targets have proven to be a promising anti-AF strategy (Voigt et al., 2012; Nattel and Dobrev, 2012). In this work, slow pacing induces complex interaction of EAD–DAD in the model (Supplementary Figure S11). The complex dynamics of EADs promoting DADs are observed in mouse ventricular myocyte (Song et al., 2015), where elevation of ${\left[Ca^{2+}\right]}_{o}$ (2.7 mM) induced $Ca^{2+}$ overload and led to EADs followed by DADs. Using the rapid pacing protocol, the model demonstrated a transient phase of hypercontractility, ${\left[Ca^{2+}\right]}_{SR}$ loading, and DAD induction. The DADs appeared in the first beat after pause and were eliminated afterward because of the beat-to-beat release of ${\left[Ca^{2+}\right]}_{SR}$ load. The loading of the reticulum plays a substantial role in determining the amplitude of DADs. In experiments, a strong emphasis has been placed on the influence of ${\left[Ca^{2+}\right]}_{SR}$ load on the initiation of DADs (Fink et al., 2011). Voigt et al. (2014) found enhanced ${\left[Ca^{2+}\right]}_{SR}$ load because of increased uptake that promotes ${\left[Ca^{2+}\right]}_{SR}$ leak, causing DADs.

### 4.5 Slowing of the repolarization rate by 4-AP can eliminate phase 3 EADs

Decreased APD, rapid pacing rates, and strong ${\left[Ca^{2+}\right]}_{SR}$ release can elicit EADs immediately after the termination of AF, as observed in isolated coronary-perfused canine right atria (Burashnikov and Antzelevitch, 2003). Phase 3 EAD-induced triggered beats can reinitiate AF following termination of paroxysmal AF but not persistent AF (Burashnikov and Antzelevitch, 2006). Atrial cells can predominantly produce phase 3 EADs, with sporadic cases of phase 2 EAD development (Burashnikov and Antzelevitch, 2006; 2003). Class III anti-arrhythmic agents are clinically associated with ventricular, but not atrial, proarrhythmia. However, some patients with congenital long QT syndrome have developed brief episodes of polymorphic atrial tachyarrhythmias that resemble an atrial form of torsade de pointes (Kirchhof et al., 2003) but are less prevalent clinically (Burashnikov and Antzelevitch, 2006). Furthermore, $I_{\mathrm{KACh}}$ protects the model against phase 2 EADs by shortening the APD (see, e.g., Supplementary Figure S9). Therefore, the 4-AP-induced exacerbation of phase 2 EADs, due to ionic current variation, should be speculated to be less vulnerable to AF development and maintenance. The only potentially uncertain condition might be the concomitant block of $I_{\mathrm{Kur}}$ and $I_{\mathrm{KACh}}$ that could uncover the risk of phase-2 EADs in clusters of susceptible cells. This aspect deserves further investigation. The results of the present study demonstrate that repolarization abbreviation may contribute to arrhythmogenesis by facilitating the development of phase 3 EADs. In particular, $I_{\mathrm{KACh}}$ is necessary to induce phase 3 EADs by increasing the repolarization rate. In fact, the role of $I_{\mathrm{KACh}}$ in sustaining AF is well established; it facilitates re-entry of circuits and stabilizes them, highlighting its potential for clinical applications (Mitrokhin et al., 2024). With the application of 4-AP, such EADs are eliminated due to slowed repolarization. A 4-AP-induced lengthening of APD may lengthen the ERP, which can be beneficial in disrupting the re-entrant wavelets and, therefore, the AF. Overall, in this work, we provide a comprehensive understanding of potential mechanisms and pharmacological targets in AF, which can help analyze cardiac arrhythmias.

## 5 Potential limitations

Some limitations should be highlighted. We have not simulated the state dependence and use dependence of the $K^{+}$ -channel block in evaluating a realistic compound. This would require the availability of data regarding binding kinetics, drug-binding mechanisms, and the affinity of drug compounds. In this study, we have simulated a single-cell electromechanical model, with no spatiotemporal details of the dyadic space and $Ca^{2+}$ handling, that is, spatial distribution of a network of $Ca^{2+}$ release units (CRUs). The placement of the L-type $Ca^{2+}$ channel in close vicinity of CRUs can simulate the effect of $Ca^{2+}$ waves emanating from various subcellular regions and integrating to generate DADs (Mitrokhin et al., 2024; Blatter, 2017). Moreover, using a common-pool model of $Ca^{2+}$ cycling, we cannot quantify the actual magnitude of DADs in real atrial cells; therefore, in this study, the DADs induced were quite small in amplitude. Protein kinase A (PKA) signaling activation by isoprenaline has emerged as a transducer to increase arrhythmia propensity. The PKA phosphorylation can be included as a future direction of this study. The scope of this study was limited to a single cell; thus, a future study would be needed to determine the $K^{+}$ -channel block response on AF reinitiation by running 2D or 3D tissue-level simulations.

## 6 Conclusion

In this work, we have analyzed the response of the $K^{+}$ -channel block on AP abnormalities induced by the bidirectional electromechanical coupling. We have determined the underlying mechanisms by which the low-dose $K^{+}$ -channel block can reverse the arrhythmogenic effect of AF. Moreover, we have highlighted potential pharmaceutical targets that could help reduce the AF burden. We have identified a dual response of 4-AP on the arrhythmogenic substrate: it exerted proarrhythmic effects in the context of EADs driven by AP– $Ca^{2+}$ forward coupling while exhibiting an anti-arrhythmic response against abnormalities arising from the $Ca^{2+}$ –AP feedback, including RyR-sensitized EADs, DADs triggered by cytosolic $Ca^{2+}$ , and EADs associated with the transient phase of hypercontractility. Desensitization of myofilaments can eliminate AP– $Ca^{2+}$ -induced EADs under AF conditions, but not under SR conditions. The atrial selective $K^{+}$ -channel block, therefore, has much potential to produce favorable anti-arrhythmic effects against AF arrhythmogenesis driven by $Ca^{2+}$ –AP coupling only. This work can be extended to the multicellular tissue scale to examine the role of $K^{+}$ channel blockade in terminating reentrant excitation waves.

## Data Availability

The original contributions presented in the study are included in the article/Supplementary Material, further inquiries can be directed to the corresponding author.
