# Supplementary material for: K+ channel blockade limits AF and suppresses phase 3 EADs by slowing repolarization in an electromechanical cell computational model
Source: Front Physiol. 2026 Jan 22;16:1704051. doi: 10.3389/fphys.2025.1704051 (PMC12872496; doi:10.3389/fphys.2025.1704051)
Supplement: Supplementary file 1 [file DataSheet1.pdf]

Supplementary Material

1 SUPPLEMENTARY TABLES AND FIGURES

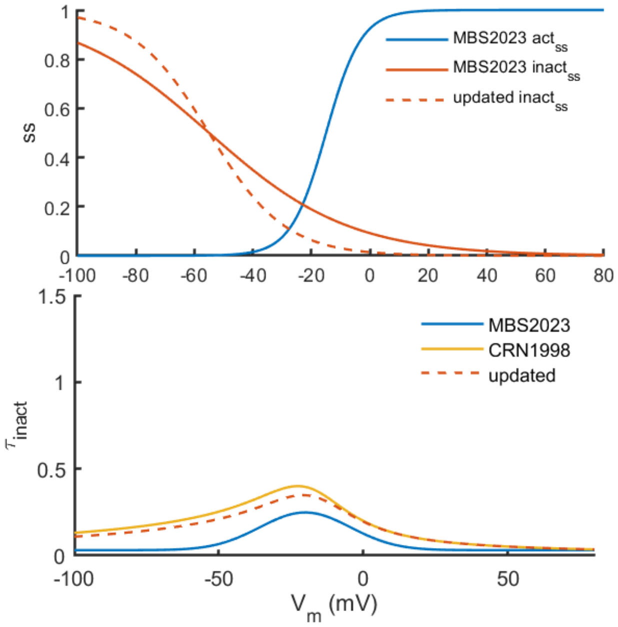

Figure S1. Updated steady-state activation (in blue) and inactivation curve (in red dashed line) for I_{Kr} current in comparison with MBS2023 model (in red solid line). Bottom panel: the updated time constant curve (in red dashed line) is compared with MBS2023 version (in blue) and Courtemanche1998 (in yellow) models.

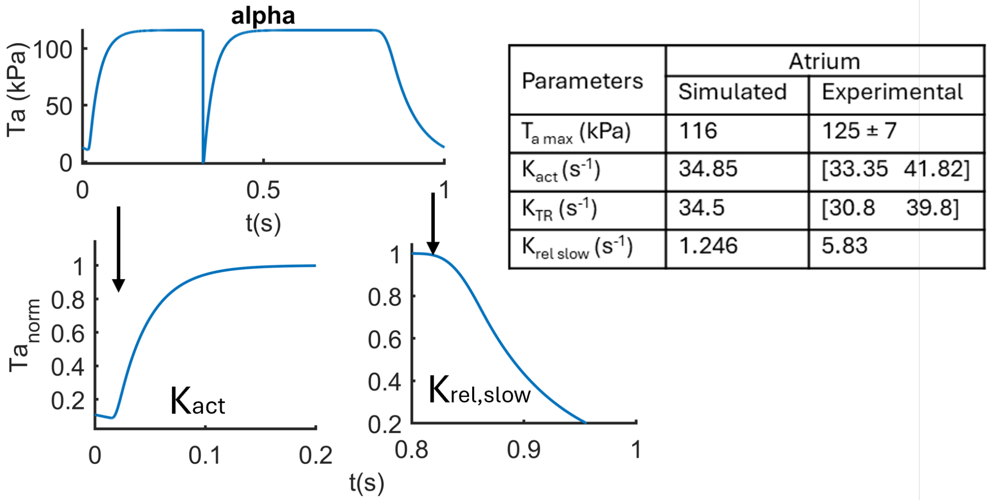

Parameters	Atrium	
	Simulated	Experimental
$T_{a\,max}$ (kPa)	116	125 ± 7
K_{act} (s^{-1})	34.85	[33.35 41.82]
K_{TR} (s^{-1})	34.5	[30.8 39.8]
$K_{rel\,slow}$ (s^{-1})	1.246	5.83

Figure S2. Force developed using fast solution switching protocol for human atrial myosin isoform expressing alpha myofibrils. The protocol was run for 1sec, where the arrows indicate the time of switching the pCa levels. The force development phase estimates of K_{act}/K_{tr}, and the relaxation phase determines $K_{rel,slow}$. The force transition rates obtained by model parameter tuning in comparison to the experimental data (Piroddi et al., 2007).

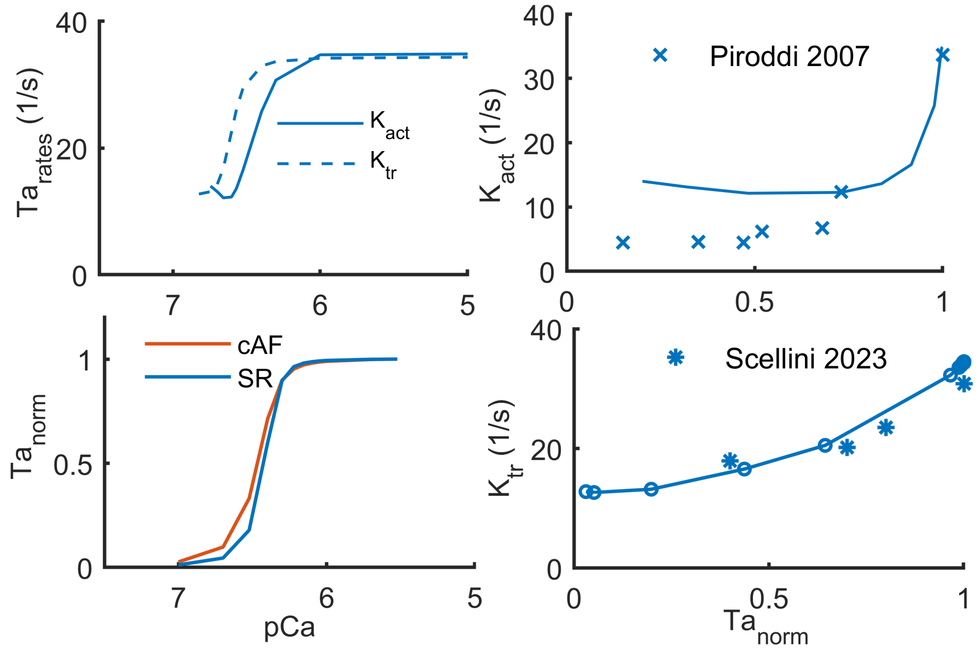

Figure S3. Calibration of contraction parameters based on force development transition rates K_{act} and K_{tr} extracted from using fast solution switching protocol. Using the protocol, with varying Ca^{2+} pulses, K_{act} and K_{tr} were fit on the data ((Piroddi et al., 2007) in the cross and (Scellini, 2024) in the asterisk, respectively) as shown in the right panels. The Ta-pCa curves used in the model under SR (in blue), and cAF condition (in red) are shown on bottom left panel.

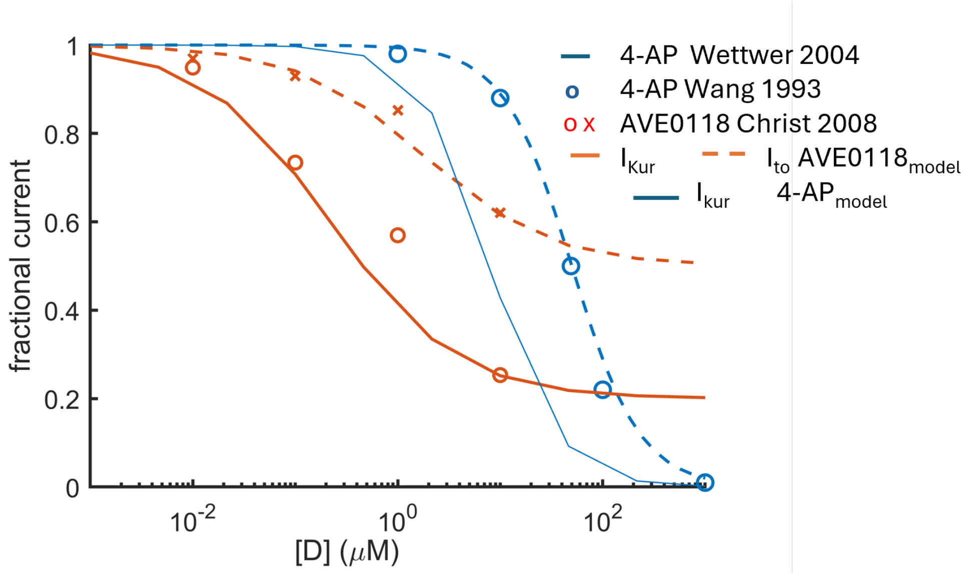

Figure S4. Dose-response curves for modelling K^{+}-channels, I_{Kur} (in open circles), and I_{to} (in cross) using pore block scheme based on experimental data (Wettwer et al., 2004; Wang et al., 1993) for 4-AP (in blue), and (Christ et al., 2008) for AVE0118 (in red).

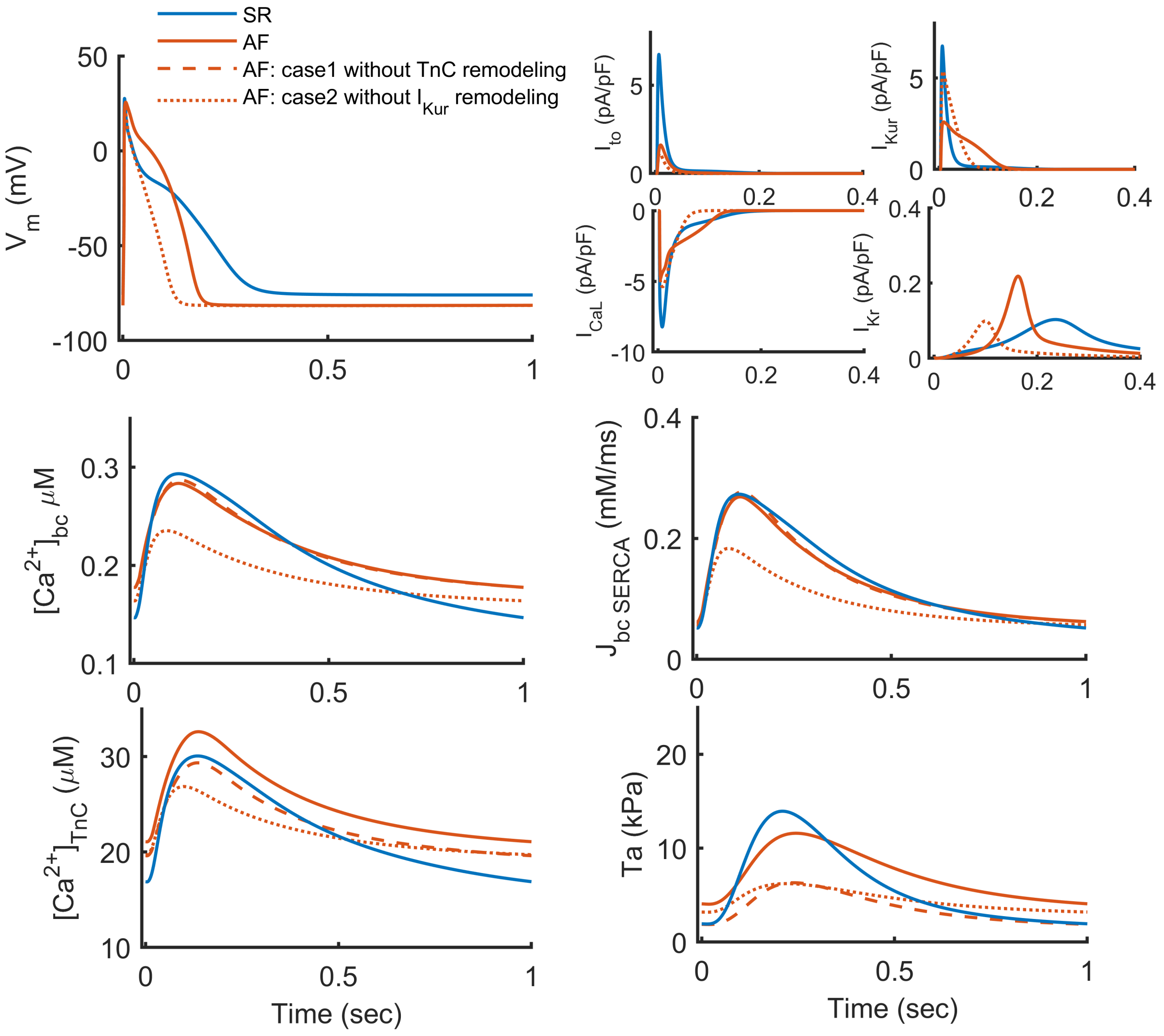

Figure S5. Characteristics of updated version of MBS2023 in SR (in blue) and cAF (in red) condition. Two cases under AF: case1 AF condition without contractile remodeling (TnC remodeling) in dashed red line, case2: AF condition without I_{Kur} remodeling. AP shapes with ionic currents I_{to}, I_{Kur}, I_{CaL}, and I_{Kr} are shown in the top panels. Ca^{2+}-transient in the bulk compartment (bc) (middle left), SERCA uptake flux from bc to SERCA (middle right), amount of Ca^{2+}- concentration bound to troponin ($[Ca^{2+}]_{TRPN}$) (bottom left), and the active force Ta (bottom right).

Table S1. Parameters modified in the updated MBS2023 model in comparison to the original model.

	Parameters	MBS2023	Updated MBS2023
1	• I_{to} formulation<br>• G_{to}	• Based on α-subunit encoded Kv1.4 gene expressed in rabbits.<br>• 8.175 nS	• Updated to human atrial isoform Kv4.3<br>• 12.25nS
2	• I_{Kr} inactivation gate<br>• G_{Kr}	• Slope =24<br>• 0.5nS	• Slope = 13. Increased rectification of the steady state I-V relation (Fig. S1).<br>• Update (a) results in a reduced window current therefore, to restore the current amplitude, it was increased by a factor of 4 i.e. 2nS
3	G_{Na}	340mS	250mS
4	Half value of I_{CaL} CDI gate	0.65μM	0.68μM The changes induced by the above 1-3 parameters resulted in an AP shape with a sustained plateau phase that allows more Ca^{2+} to enter the cell. Therefore, to avoid the reopening of the current gate, we shifted the half value of the CDI to a slightly higher Ca^{2+} level.
5	Contractility-related parameters reference to Table 1 in the main manuscript	Default RDQ2020 model with slow thin and fast thick filament kinetics.	Updated the thin filament kinetics (RU kinetics), F-pCa curve (RU steady state), thick filament kinetics (XB kinetics) based on the experimental data as shown in Table 1.
6	• RyRss recovery from inactivation<br>• Adaptation gates minimum<br>• Adaptation gates maximum	• 450msec	• 12mesc. An acceleration of thin filament kinetics has sped up the Ca^{2+}-transient decay time, which was previously quite slow in the MBS2023 model (Table 2, (Mazhar et al., 2024)). Accordingly, we accelerated the RyR recovery from inactivation time, which had been slowed to accommodate a slower Ca^{2+}-transient.<br>• Additionally, we restored the original adaptation variable values to those used by the parent model, Koivumaki (2011).
7	fCaNX from I_{NaCa} current	1	1.3, To increase the inward mode of the current.

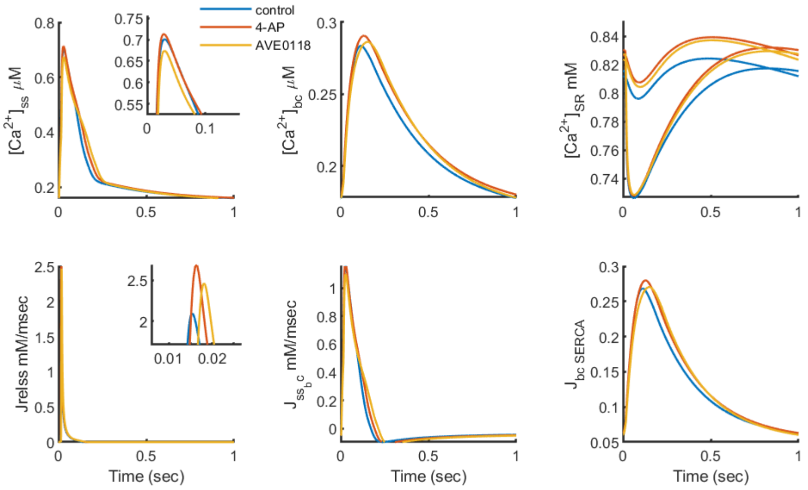

Figure S6. K^+-channel block drugs 4-AP (5μM) (in red), AVE0118 (6 μM) (in yellow) response on Ca^{2+}-handling, compared under control condition (in blue). $[Ca^{2+}]_{ss}$ is in the subspace (top left panel), $[Ca^{2+}]_{bc}$ in the bulk compartment (top middle panel), $[Ca^{2+}]_{SR}$ is in the SR store for two subcompartments (top right panel), J_{relss} is the release flux in the ss (bottom right panel), J_{ss-bc} is the diffusion from *ss* to *bc* (bottom middle panel), $J_{bc-SERCA}$ is the flux from *bc* to SERCA (bottom right panel).

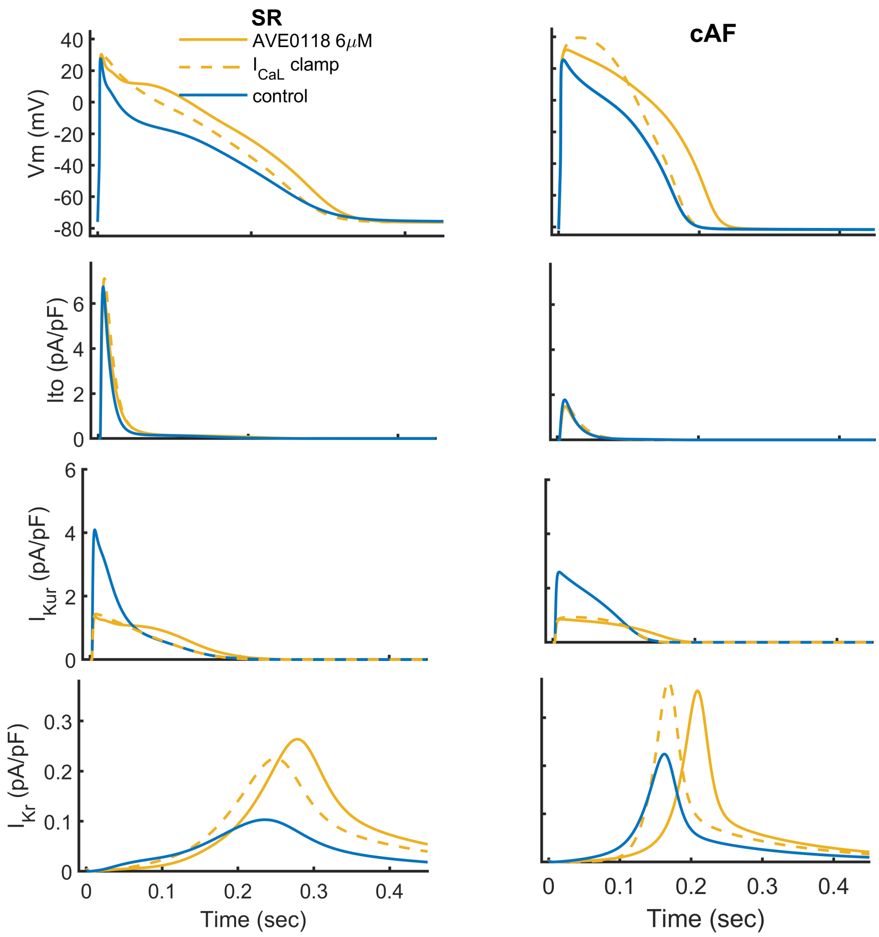

Figure S7. The effect of AVE0118 on action potential under I_{CaL} clamped to control waveform for both SR (left column), cAF (right column). The corresponding ionic currents- I_{to} (second row), I_{Kur} (third row), and I_{Kr} (bottom row) are shown for comparison.

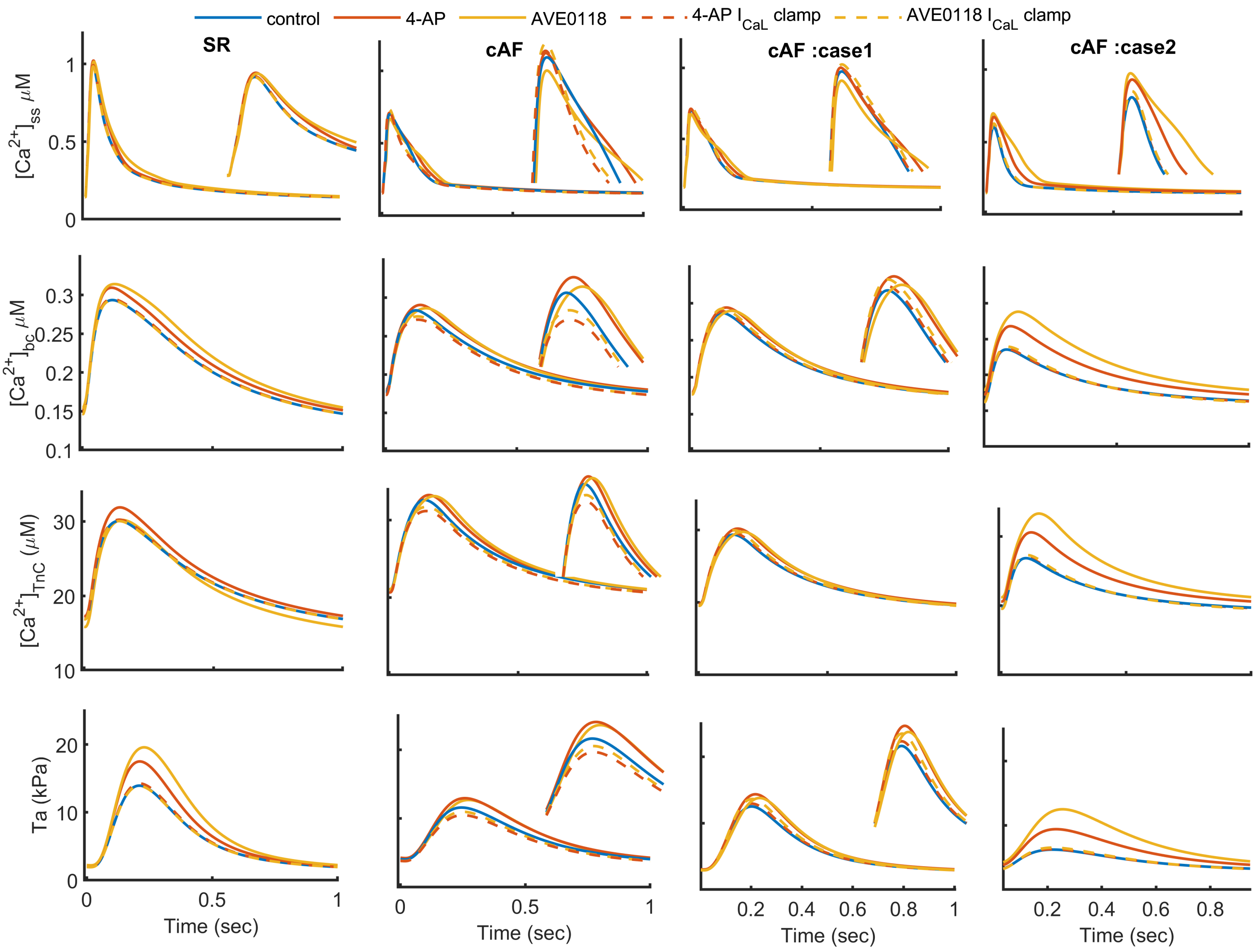

Figure S8. Ca^{2+}-transient and contractile force under I_{CaL} clamp condition in SR (column 1), cAF (column 2), cAF: case1 (column 3) without I_{Kur} AF-induced remodeling, and cAF: case 2 (column 4) without contractile remodeling.

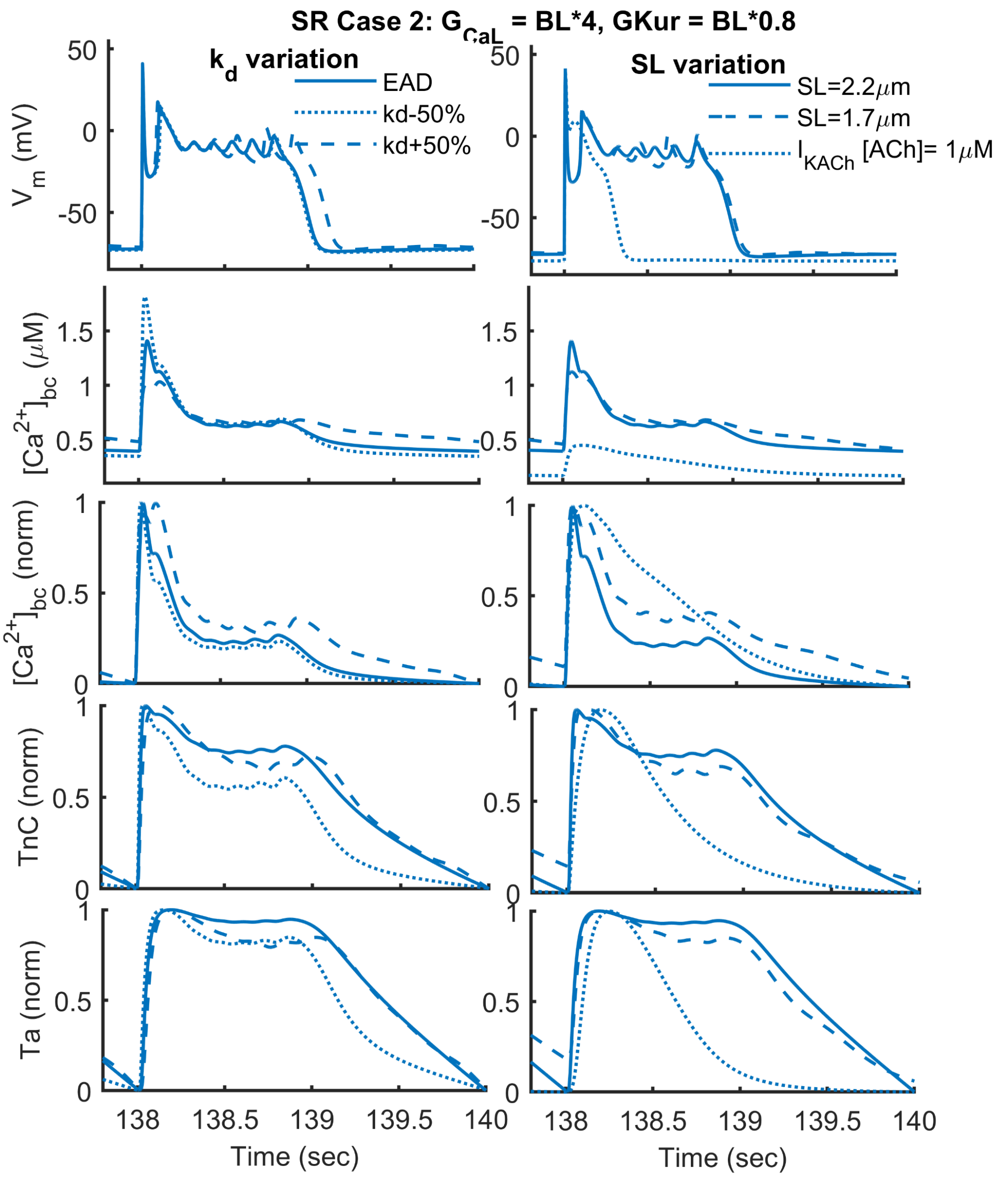

Figure S9. Role of myofilament sensitivity (kd) and length (SL) variation on phase 2-EAD under SR condition. The presence of acetylcholine-activated outward potassium (I_{KACh}) current with [Ach]=1μM can abbreviate the AP, thereby eliminating the EADs (in dotted line).

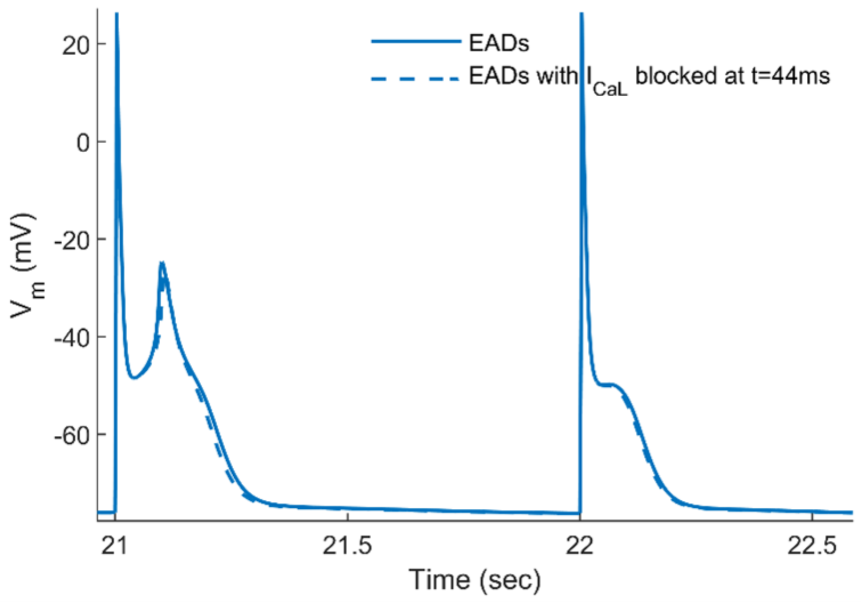

Figure S10. Block of I_{CaL} current just after the time, t=44msec when the take-off potential arrives. Block of I_{CaL} current does not contribute to the phase 3-EAD initiation.

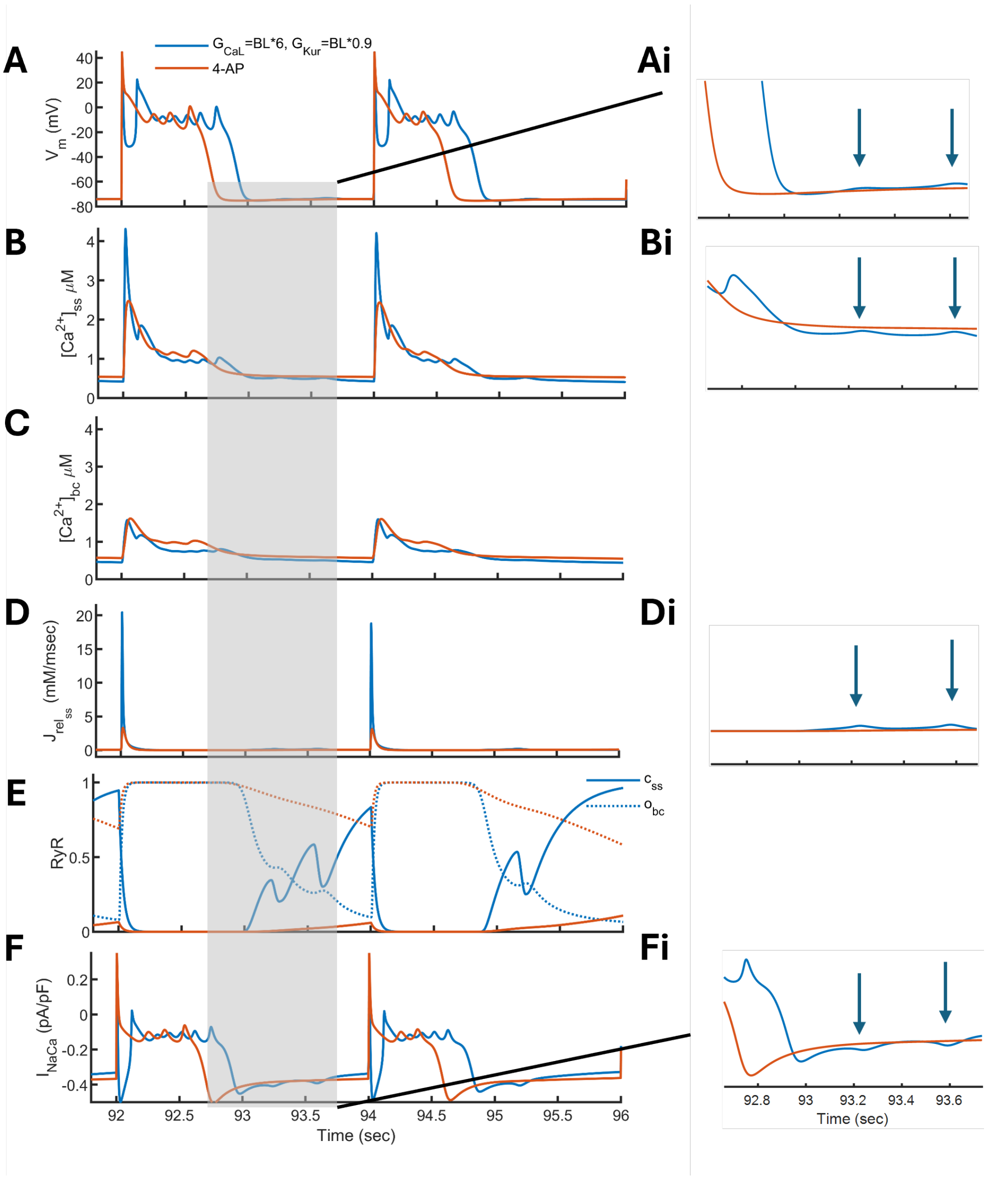

Figure S11. Development of delayed afterdepolarizations (DADs) in the updated MBS2023 model (in blue) and its response in the presence of 4-AP (in red). DADs induction using slow pacing at BCL 2sec for 70 beats. A) the action potential Vm, B) Ca^{2+}-transient in the subspace $[Ca^{2+}]_{ss}$, C) and $[Ca^{2+}]_{bc}$ in *bc* compartment, D) release flux J_{relss}, E) RyR activation in *bc* (obc) (in dotted line), and inactivation in *ss* (css) (in solid line), F) I_{NaCa} current. All the panels are zoomed in time from 92.8 to 93.6 sec in the inset from Ai to Fi.

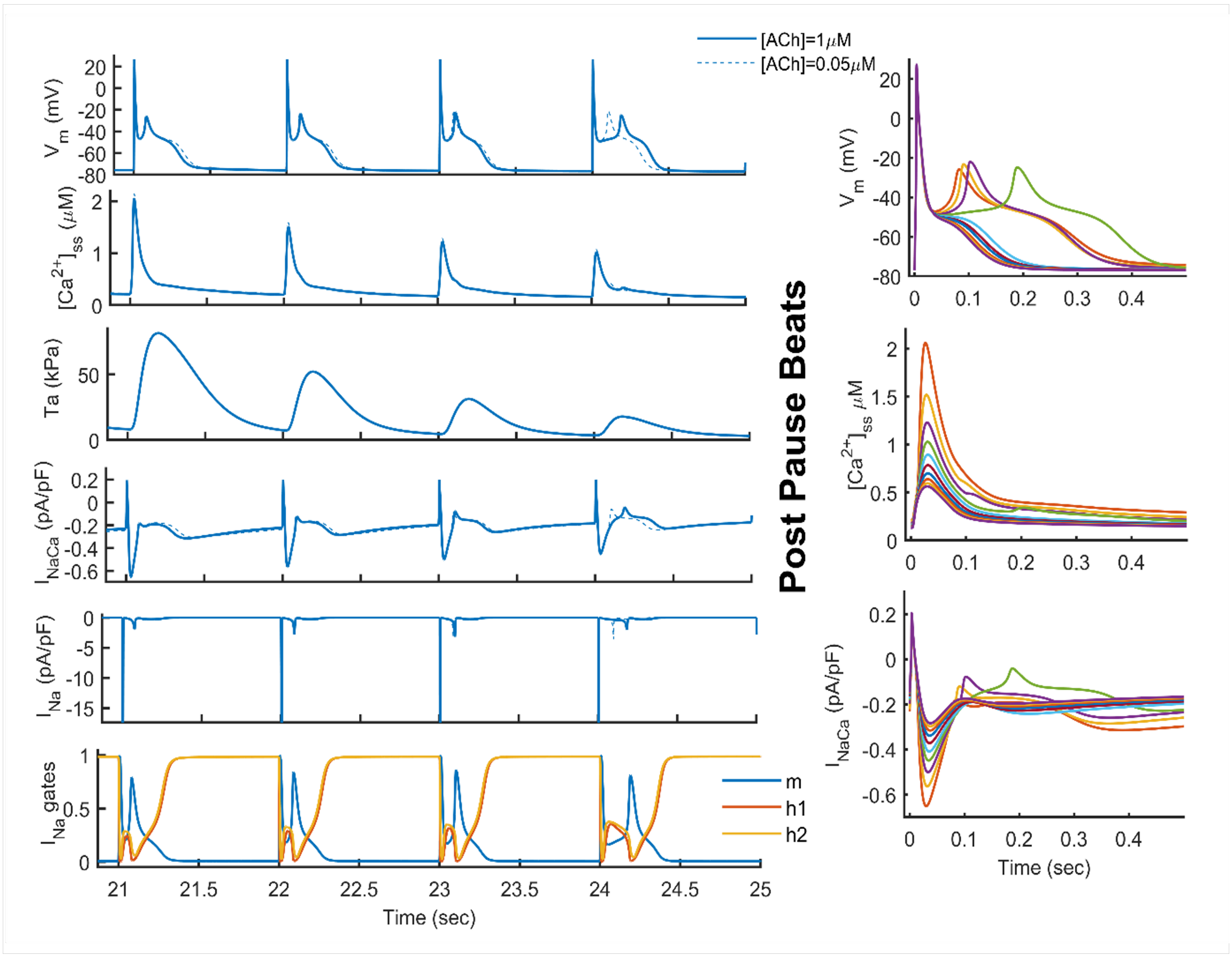

Figure S12. Role of varying acetylcholine-activated potassium current I_{KACh} amplitude on phase 3 EADs. I_{KACh} is reduced to half from the baseline BL value resulting in increased propensity towards phase-3 EADs elucidation. With reduced I_{KACh} amplitude and concentration [ACh]=0.05 (in dashed line), they are sensitive to EADs initiation in the model.

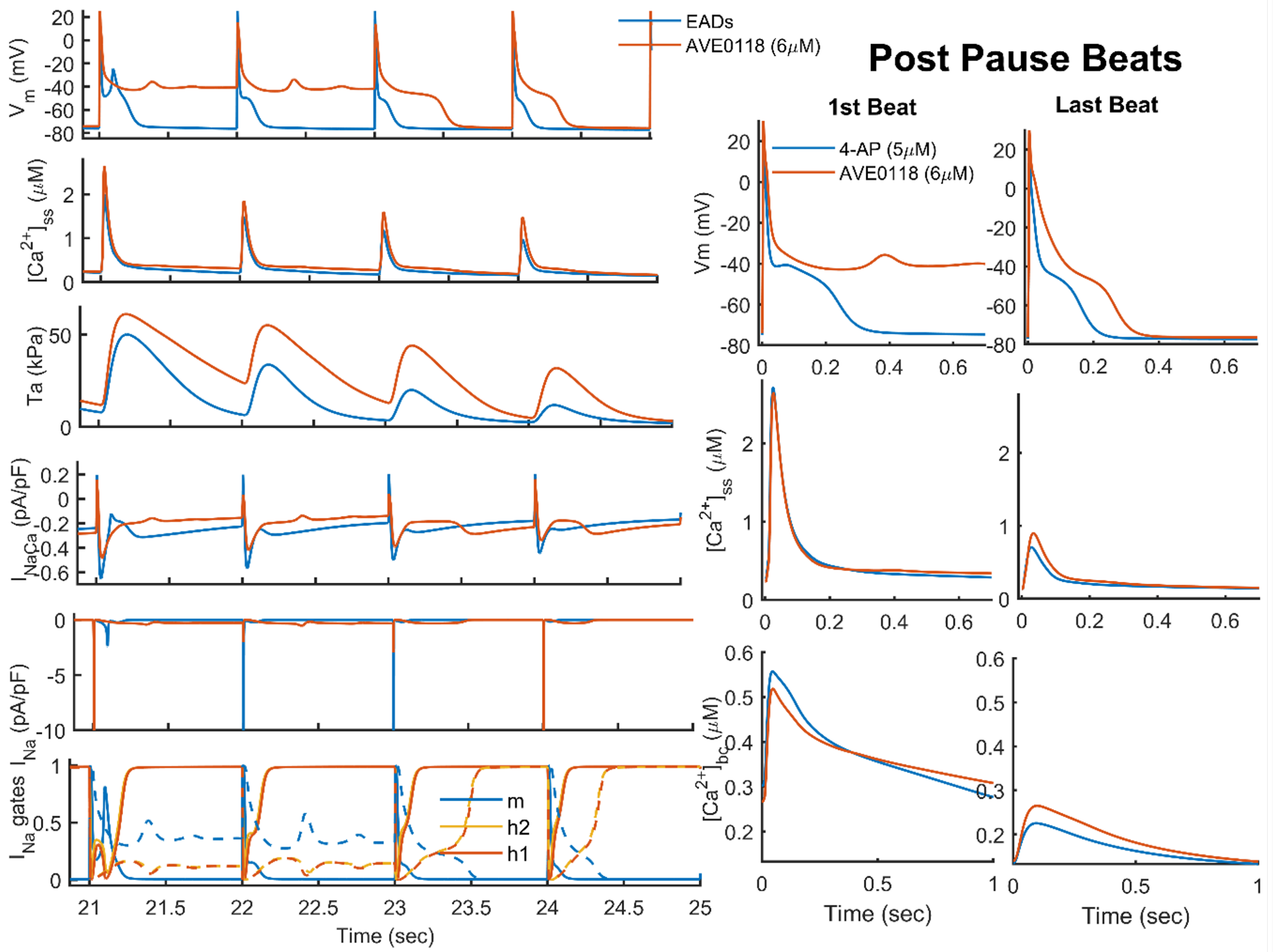

Figure S13. AVE0118 (6μM) response on phase-3 EADs in human atrial electromechanical model. The model was rapidly paced at 10Hz for 20sec and returned to sinus rhythm after a pause in the presence of I_{KACh}. The transient rise of Ca^{2+} in the cytosol (Ca_{ss}) increases the inward mode of I_{NaCa} current. A parallel rise in bulk Ca^{2+} results in transient hypercontractility. A strong I_{NaCa} creates a transient prolongation of APD and facilitates the reactivation of I_{Na} current. Post-pause beats superimposed (on right) show a beat by beat reduction in SR Ca^{2+} loading, reverses the APD prolongation (Aii), Ca^{2+} accumulation (Bii and Cii).

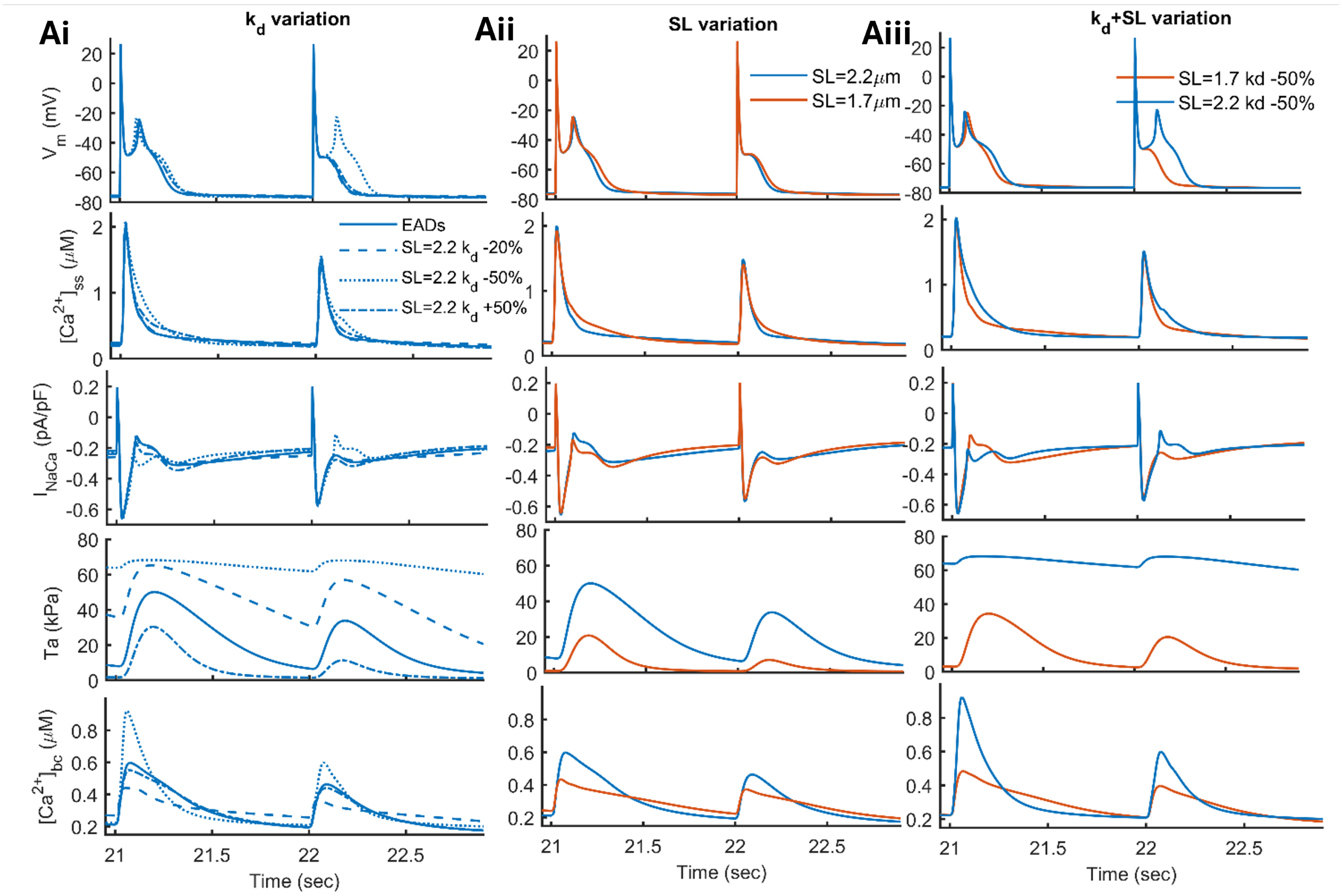

Figure S14. Myofilament sensitivity (kd) and length (SL) variation and its effect on phase 3 EADs related vulnerability. Sensitization of myofilament (kd-50% in dotted line) can induce phase 3 EADs in more than one subsequent beat after the pause. Short sarcomere can reduce the affinity of Ca^{2+}-TnC bound, hence reducing the hypercontractility phase in the model.

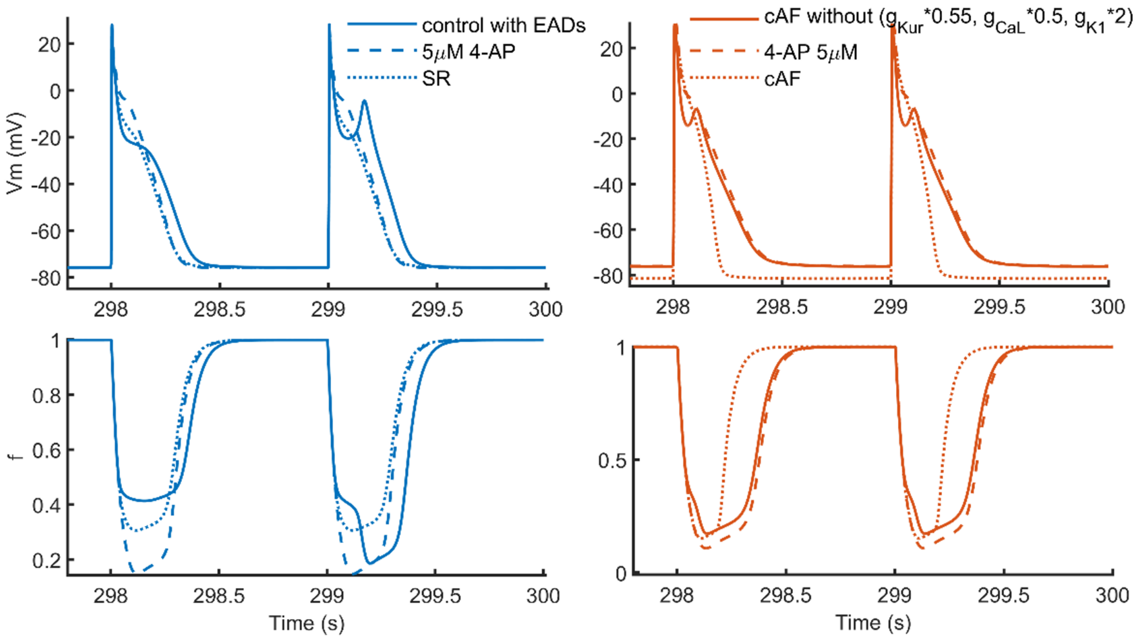

Figure S15. EADs induced by RyR sensitization and its response in the presence of 4-AP under SR (on left in blue), and AF (on right in red) conditions. The panel below shows the inactivation gate time course in control (dotted line), control with EADs (solid line), and in the presence of 4-AP (dashed line).

REFERENCES

Christ, T., Wettwer, E., Voigt, N., Hala, O., Radicke, S., Matschke, K., et al. (2008). Pathology-specific effects of the ikur/ito/ik, ach blocker ave0118 on ion channels in human chronic atrial fibrillation. *British journal of pharmacology* 154, 1619–1630

Mazhar, F., Bartolucci, C., Regazzoni, F., Paci, M., Dedè, L., Quarteroni, A., et al. (2024). A detailed mathematical model of the human atrial cardiomyocyte: integration of electrophysiology and cardiomechanics. *The Journal of physiology* 602, 4543–4583

Piroddi, N., Belus, A., Scellini, B., Tesi, C., Giunti, G., Cerbai, E., et al. (2007). Tension generation and relaxation in single myofibrils from human atrial and ventricular myocardium. *Pflügers Archiv-European Journal of Physiology* 454, 63–73

[Dataset] Scellini, B. (2024). Mavacamten depresses human atrial contractility

Wang, Z., Fermini, B., and Nattel, S. (1993). Delayed rectifier outward current and repolarization in human atrial myocytes. *Circulation research* 73, 276–285

Wettwer, E., Hála, O., Christ, T., Heubach, J. F., Dobrev, D., Knaut, M., et al. (2004). Role of I Kur in Controlling Action Potential Shape and Contractility in the Human Atrium Influence of Chronic Atrial Fibrillation. *Circulation* , 2299–2306
